# Mapping the neuroimaging landscape of inflammatory bowel disease: a bibliometric analysis and systematic scoping review

**DOI:** 10.3389/fnins.2026.1821831

**Published:** 2026-06-04

**Authors:** Yingchun Zhang, Liyuan Gao, Jingjing Cao, Tingting Zhang, Xiaoyu Wang

**Affiliations:** 1 Department of Gastroenterology, Zhangjiagang Traditional Chinese Medicine Hospital Affiliated to Nanjing University of Chinese Medicine, Suzhou, China; 2 Department of Pharmacy, Zhangjiagang Traditional Chinese Medicine Hospital Affiliated to Nanjing University of Chinese Medicine, Suzhou, China; 3 Department of Neurology, First Affiliated Hospital of Dalian Medical University, Dalian, China

**Keywords:** inflammatory bowel disease, neuroimaging, bibliometric analysis, brain-gut axis, functional magnetic resonance imaging

## Abstract

**Background:**

Inflammatory bowel disease (IBD) is recognized as a prototypical disorder of brain-gut interaction. Although neuroimaging research in this field has advanced rapidly in recent years, the findings remain fragmented across multiple disciplines, and a systematic integration of the literature is lacking.

**Objective:**

This study presents the first integrated bibliometric analysis and literature review to map the landscape and evolving trends of neuroimaging research in IBD over the past two decades and to identify the knowledge base and research frontiers.

**Methods:**

We conducted a systematic search of the Web of Science Core Collection and Scopus databases for IBD-related neuroimaging literature published between January 2000 and January 2026. Following the PRISMA guidelines, two independent reviewers screened titles, abstracts, and full texts. A total of 175 articles met the inclusion criteria. Data were extracted on study characteristics, neuroimaging modalities, and clinical findings. For the synthesis, we employed a dual approach: (1) a bibliometric analysis using VOSviewer, Biblioshiny, and CiteSpace to map publication trends, collaboration networks, and research hotspots; and (2) a structured literature review across five predefined dimensions: technical modalities, brain region–symptom associations, subtype differences, mechanistic pathways, and clinical translation.

**Results:**

The systematic search and selection process identified 175 articles for final synthesis. The field has entered a phase of rapid expansion since 2021, with China and the United States as core contributing countries. Emerging frontiers include the “brain-gut axis” and the “default mode network.” The literature synthesis indicates that: (1) brain alterations are predominantly localized within an emotional and interoceptive network (anterior cingulate cortex, insula, and amygdala), with abnormalities generally associated with abdominal pain, anxiety, and depression; and (2) Crohn’s disease and ulcerative colitis appear to exhibit distinguishable neuroimaging phenotypes, though direct comparative studies remain limited.

**Conclusion:**

This study systematically clarifies the knowledge structure of the IBD neuroimaging field, demonstrates that the available neuroimaging evidence is consistent with the brain-gut axis as a central theoretical framework, and identifies subtype-specific neural characteristics. Future efforts should prioritize large-sample multicenter validation, longitudinal designs capable of testing mechanistic hypotheses, and multimodal data integration to transition the field from descriptive observations toward clinically meaningful applications,though substantial barriers—including small sample sizes, methodological heterogeneity, and lack of standardization—must first be overcome.

## 1 Introduction

Inflammatory bowel disease (IBD), primarily comprising Crohn’s disease (CD) and ulcerative colitis (UC), represents a group of chronic relapsing gastrointestinal inflammatory conditions of unknown etiology (Lv et al., 2017; Yin et al., 2026). Historically, clinical management and research have focused predominantly on localized intestinal inflammation, associated complications, and immunomodulatory therapies. Nevertheless, a substantial proportion of IBD patients experience systemic symptoms including depression, anxiety, severe fatigue, cognitive impairment, and widespread abdominal pain (Bisgaard et al., 2022; Ge et al., 2022). These manifestations cannot be fully explained by the degree of intestinal inflammation and often respond poorly to conventional anti-inflammatory treatments (Person and Keefer, 2021), suggesting the involvement of complex pathophysiological mechanisms independent of peripheral inflammation—factors that have emerged as critical determinants of patient quality of life.

This clinical challenge has prompted a paradigm shift in how IBD is conceptualized: rather than being viewed as a disease confined to the gut, IBD is now recognized as a prototypical disorder of brain-gut interaction (Bonaz and Bernstein, 2013). The brain-gut axis theory provides the central explanatory framework, emphasizing bidirectional communication between the brain and the gastrointestinal tract through neural, endocrine, and immunological pathways (Lv et al., 2017; Person and Keefer, 2021; Yin et al., 2026). Elucidating the characteristics of brain function alterations in patients with IBD and understanding the patterns of association between intestinal inflammation and the central nervous system are therefore essential for overcoming current clinical bottlenecks and advancing toward personalized precision medicine (Silva et al., 2024).

Advances in neuroimaging technology, particularly functional magnetic resonance imaging (fMRI), have provided powerful tools for visualizing these previously hidden brain-gut interactions. Accumulating evidence has revealed alterations in gray matter volume, white matter microstructural abnormalities, and altered functional connectivity in brain regions involved in pain processing, emotional regulation, cognition, and interoception—including the anterior cingulate cortex (ACC), insula, hippocampus, and default mode network (DMN)—in patients with IBD (Kong et al., 2021; Yang et al., 2024b; Yin et al., 2026). However, current research findings are broadly disseminated across multidisciplinary literature spanning gastroenterology, neuroscience, psychiatry, and related fields. Moreover, substantial heterogeneity exists in study designs, neuroimaging techniques, and outcome measures, contributing to inconsistent conclusions (Silva et al., 2024). Traditional narrative reviews cannot objectively quantify the overall landscape of the field, nor can they systematically identify the knowledge base or track the evolution of research hotspots. To date, an integrated approach combining “macro-level mapping” with “micro-level synthesis” remains lacking.

To address this gap, we conducted an integrated bibliometric analysis and literature review to map the landscape and evolving trends of neuroimaging research in IBD. By combining science mapping with thematic synthesis, this study aims to: (1) delineate academic output trends and identify core contributors, key research themes, and technical hotspots; (2) synthesize the knowledge base, clinical correlates, and subtype-specific neuroimaging features; and (3) detect research frontiers and potential future directions. The anticipated results will provide a comprehensive “academic roadmap” for the field, assisting researchers in clarifying the current state of knowledge and identifying collaboration opportunities, while also offering a reference for advancing neuroimaging techniques in IBD brain-gut axis research and highlighting the barriers that must be addressed before clinical translation can be meaningfully pursued.

## 2 Methods

### 2.1 Data sources and retrieval strategy

This study adopted combined retrieval from the Web of Science Core Collection (WOSCC) and Scopus databases to support both bibliometric analysis and literature review (Kumpulainen and Seppänen, 2022). By integrating their advantages of structured citation systems and broad interdisciplinary coverage (Mongeon and Paul-Hus, 2016), this approach systematically reflects the research progress in the field of IBD neuroimaging. Meanwhile, relevant clinical trial data were obtained from the PubMed database. The retrieval focused on the two core themes of “IBD” (encompassing its main disease subtypes, CD and UC) and “neuroimaging,” with a restriction to cranial imaging. Boolean logic and wildcards were used to combine relevant terms, and all relevant term variants were covered as much as possible to ensure comprehensive retrieval without omission. The retrieval scope was uniformly limited to the TOPIC field to enhance thematic relevance. The retrieval time span was from 2000 to 2026, with document types restricted to Article and Review, and language restricted to English. The retrieval cut-off date was January 27, 2026. Detailed search strategies are provided in Appendix 1.

### 2.2 Literature screening and data cleaning

A total of 1841 articles were initially retrieved (348 from WOSCC and 1,493 from Scopus). Full-record data, including titles, authors, institutions, countries, publication years, abstracts, keywords, and references, were downloaded in plain text and CSV formats. Python 3.11 was used to convert the CSV format of Scopus data into the same plain text format as WOSCC (Diez-Junguitu and Peña-Cerezo, 2026). Subsequent data cleaning was conducted with the following specific steps: deduplication based on DOI, exclusion of records with “[Anonymous]” in the author field, removal of virtual institutions, and merging and standardization of duplicate institutional names. After cleaning, 1,508 articles remained (336 from WOSCC and 1,172 from Scopus).

Two independent researchers conducted dual-screening at each stage to minimize bias: 1. Initial screening: Review of titles and abstracts against pre-defined criteria; 2. Full-text screening: For articles meeting initial screening criteria, full-texts were retrieved to confirm eligibility. Any disagreements between the two researchers were resolved through discussion or, when necessary, consultation with a third senior researcher to reach consensus in accordance with the Preferred Reporting Items for Systematic Reviews and Meta-Analyses (PRISMA) guidelines (Page et al., 2021). Inclusion criteria were as follows: (1) Research subjects were human IBD patients (CD or UC) or IBD animal models; (2) Research methods involved *in vivo* brain neuroimaging scanning and analysis (including structural, functional, or molecular neuroimaging). Exclusion criteria were as follows: (1) Research subjects were gastrointestinal diseases other than IBD or other systemic diseases; (2) The target organ of imaging was not the brain (e.g., imaging only targeting the intestines or joints); (3) Case reports that did not involve the assessment of brain structure and function and only used imaging as a diagnostic tool for neurological complications or drug side effects. Finally, 175 articles were included for both bibliometric analysis and literature review, and an additional 7 clinical trials from PubMed were exported in standardized format for integrated thematic analysis.

### 2.3 Data analysis and visualization

The final dataset of the 175 included articles was re-exported as plain text in the format of “full records and cited references” for subsequent bibliometric analyses. This study comprehensively employed multiple bibliometric software and methods, as detailed below:

VOSviewer (v1.6.20) (van Eck and Waltman, 2010), which excels in network construction and visualization, facilitates clear delineation of network structures among countries, institutions, authors, and keywords. In this study, it was primarily used to construct and visualize scientific collaboration networks (across countries, institutions, and authors) as well as keyword co-occurrence networks. Core nodes, cluster structures, and connection intensities among network elements were identified by setting appropriate co-occurrence thresholds.
Biblioshiny 5.0 (Aria and Cuccurullo, 2017) (an R package in RStudio) was applied for core bibliometric statistical analyses. Specifically, it was used to analyze annual publication trends, productivity rankings of countries, institutions, and authors, journal distribution statistics, and to characterize the macro-developmental trends of IBD neuroimaging research, providing a basis for in-depth analyses.
CiteSpace (v6.4.1) (Chen, 2006) was used to analyze the dynamic evolution of research hotspots, temporal correlations, and the field’s intellectual foundation. For burst detection and co-citation analysis, the top 20 most cited or co-cited items per time slice (two-year slices) were selected. The selection criteria included g-index (k = 25) and link retention factor (LRF = 3). This included: (1) burst detection to identify keywords and references with notable citation surges, tracing research frontier trajectories; (2) reference co-citation analysis to identify highly cited and high-centrality documents, clarifying the knowledge base and theoretical cornerstones; and (3) bibliographic coupling analysis to construct a coupling network, assess inter-document relationships, and identify core literature and key research directions.
Visualizations (including network maps and timeline maps) were generated to present results clearly and intuitively, combined with data tables. Additionally, clinical trial data retrieved from the PubMed database were organized; their basic information was sorted and classified by research topic to analyze the hot directions and potential trends of clinical research in this field.

### 2.4 Literature review methodology

To complement the macroscopic bibliometric findings with an in-depth thematic synthesis, a structured literature review was conducted on the 175 included articles and 7 clinical trials. The review comprised five core analytical dimensions: (1) Technical Modalities and Applications: We extracted and categorized the neuroimaging modalities and analytical techniques used in IBD research to summarize the technical landscape and application frequencies. (2) Brain Region–Symptom Correlates: We identified structural and functional abnormalities in key brain regions and networks, synthesizing their reported associations with core clinical symptoms, including abdominal pain, anxiety, depression, fatigue, and cognitive impairment. (3) Subtype and Stage Differences: Studies comparing CD and UC, or stratifying patients by disease activity and symptom profiles, were analyzed to delineate shared and distinct neural substrates. (4) Brain-gut Pathways: Evidence for brain-gut interaction pathways was synthesized and categorized into three primary routes—microbiota, neuroimmune, and vagal—to summarize the links between peripheral inflammation and central nervous system changes. (5) Clinical Translation and Research Gaps: We evaluated the progress of biomarker development and intervention studies, synthesizing reported limitations to identify overarching research gaps and barriers to clinical implementation. The findings were synthesized as thematic summaries, with key data presented in descriptive tables to integrate bibliometric hotspots with detailed research evidence.

### 2.5 Risk of bias assessment

Given that this is a scoping review combined with bibliometric analysis rather than an intervention-focused meta-analysis, a formal risk of bias assessment using traditional tools (e.g., Cochrane) was not applicable. Instead, we indirectly evaluated evidence quality through two approaches: (1) bibliometric indicators (e.g., citation frequency, journal impact, author network centrality) as proxies for scientific influence; and (2) systematic extraction and discussion of methodological attributes (e.g., sample size, study design) of the included studies during qualitative synthesis, with their limitations addressed in the narrative summary. Notably, we qualitatively appraised key methodological limitations across studies, including small sample sizes, cross-sectional designs, single-center settings, technical heterogeneity, and limited reproducibility, to provide a transparent evaluation of the overall evidence quality.

## 3 Results

### 3.1 Results of bibliometric analysis

#### 3.1.1 Academic output and contribution landscape

**Figure 1:**
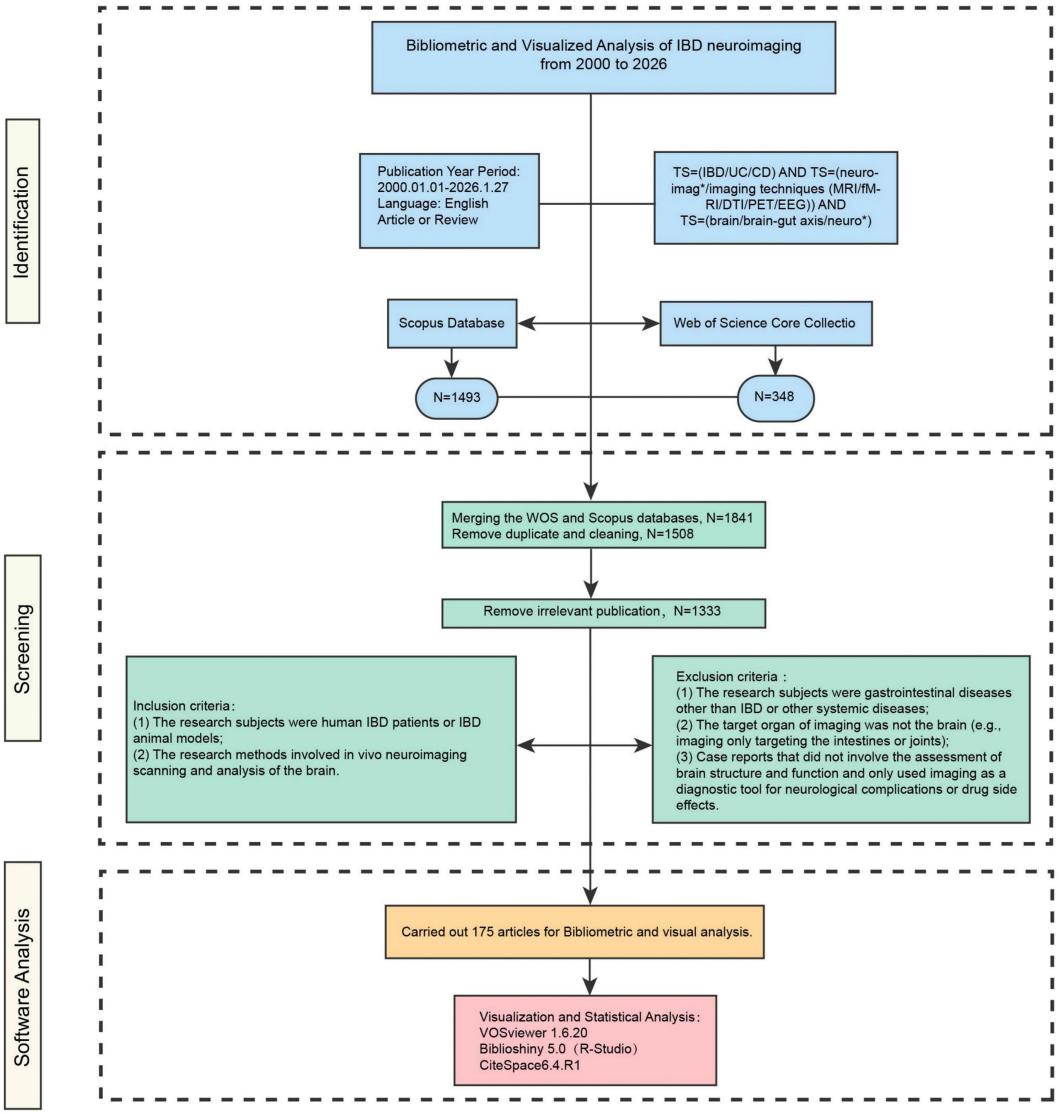
PRISMA 2020 flowchart of the literature screening process.

**Figure 2:**
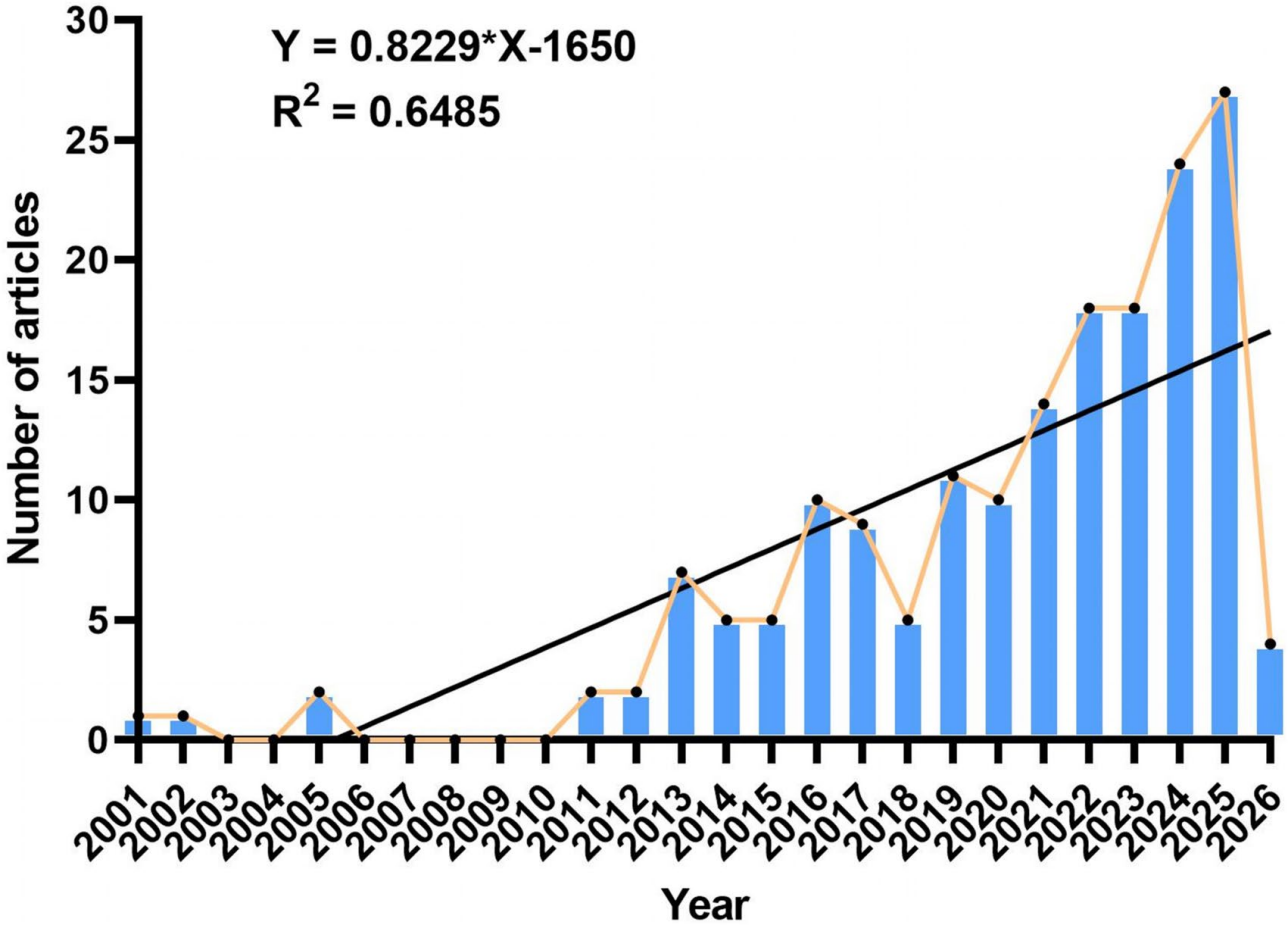
Annual publication output in IBD neuroimaging research from 2001 to 2026. Blue bars represent the number of articles published per year; the orange line depicts annual fluctuations; the black line shows the linear regression fit ( Y=0.8229X−1650 , $R^{2}=0.6485$ ). Note that 2026 data are incomplete due to the retrieval cut-off date of January 27, 2026.

Following the screening process outlined in Figure 1, the final dataset included 175 articles. The annual publication output in IBD neuroimaging research demonstrated a significant positive growth trend from 2001 to 2025, with a pronounced acceleration after 2021 (annual publications increasing from 14 in 2021 to 27 in 2025), reflecting heightened research engagement in recent years (Figure 2).

**Figure 3:**
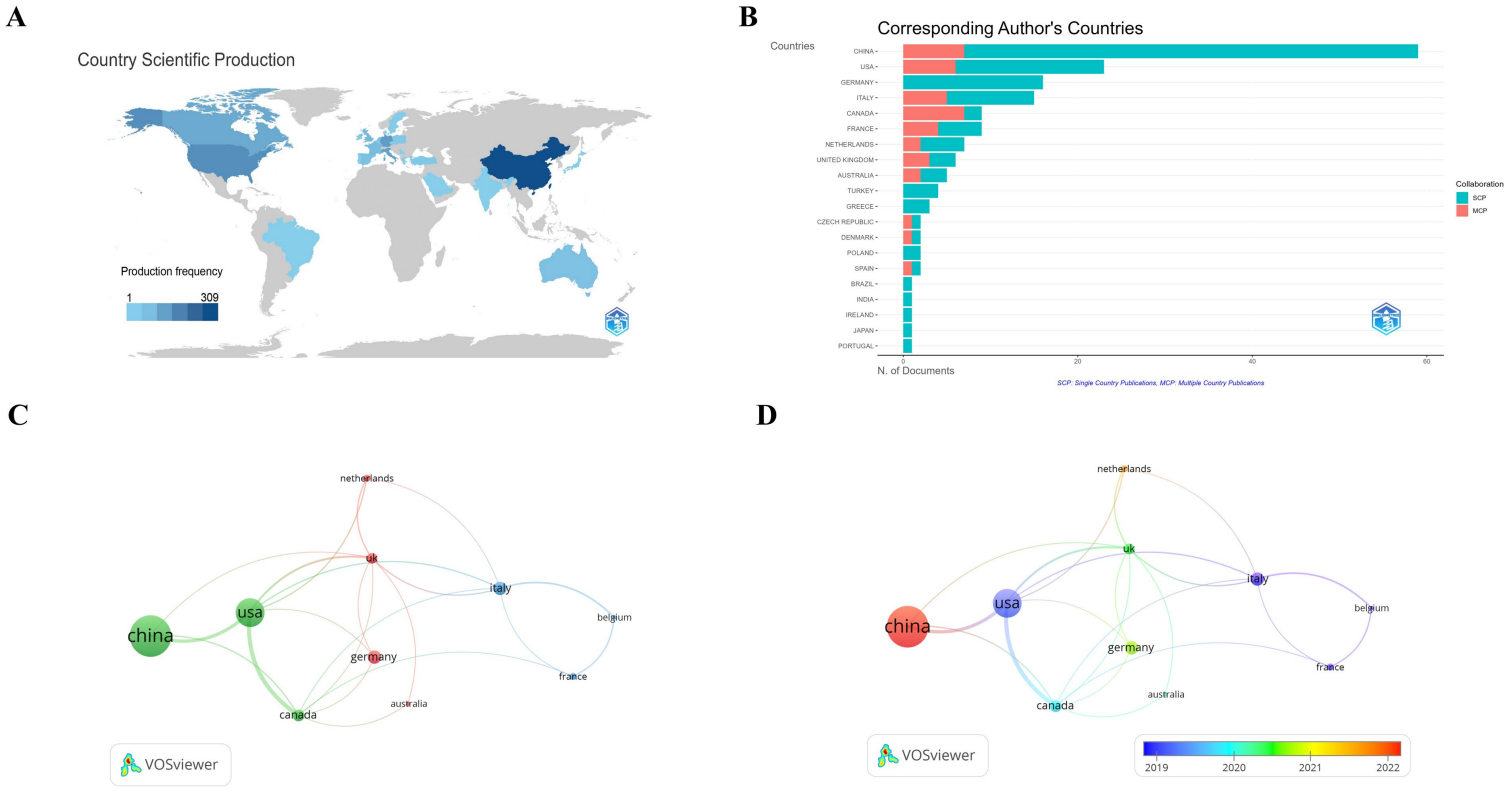
Analysis of global scientific production and collaboration in IBD neuroimaging research. **(A)** Geographical distribution of publications by country. **(B)** Corresponding authors’ countries with the highest scientific output. Bars represent the total number of publications, segmented into single-country publications (SCP, blue) and multiple-country publications (MCP, red). **(C)** Collaboration network among countries generated using VOSviewer (document threshold = 5). **(D)** Overlay visualization of the country collaboration network color-coded by average publication year.

Geographical distribution revealed a dominant role for China and the United States. China led in total output (62 documents, Table 1), while the United States, France, and Canada exhibited higher average citation impacts (France leading with 163.78, Table 1), underscoring their influential contributions. Collaboration patterns differed markedly: China’s research was predominantly domestic, whereas the United States showed extensive international collaboration (highest total link strength: 32.00). Temporal analysis indicated that China’s international collaborations intensified in recent years (2019–2022), underscoring its emergence as a key driver of global research efforts (Figure 3). This bipartite leadership, with China contributing quantitative growth and the US/Europe providing high-impact foundational insights, suggests a complementary global research ecosystem that has accelerated the field’s maturation.

**Table 1:** Top 10 most productive countries in IBD neuroimaging.

Rank	Country	Documents	Citations	Average citations	Total link strength
1	China	62	1,148	18.5	11
2	United States	42	1854	44.1	32
3	Germany	18	411	22.8	3
4	Italy	18	498	27.7	11
5	Canada	16	1,019	63.7	18
6	United Kingdom	14	867	61.9	14
7	France	9	1,474	163.8	4
8	Netherlands	9	102	11.3	6
9	Australia	5	105	21.0	2
10	Belgium	5	127	25.4	5

**Figure 4:**
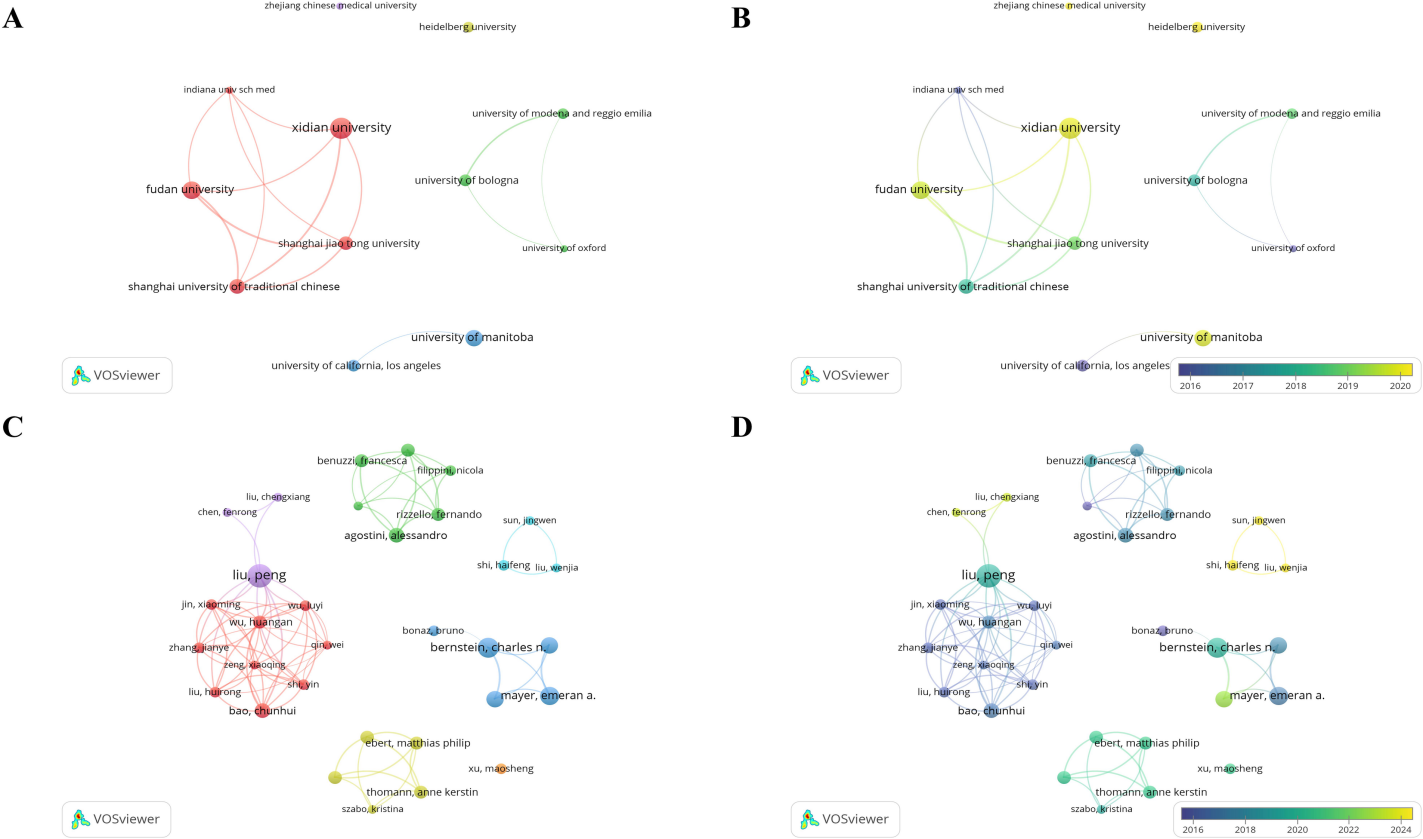
Collaboration networks of institutions and authors in IBD neuroimaging research. **(A)** Network visualization of collaborating institutions (document threshold = 5). **(B)** Overlay visualization of the institutional collaboration network color-coded by average publication year. **(C)** Network visualization of co-author relationships (document threshold = 5). **(D)** Overlay visualization of the co-author network color-coded by average publication year. All networks were generated using VOSviewer.

Institutional analysis showed that Chinese institutions dominated publication output, with Xidian University (14 documents), Fudan University (12), and Shanghai University of Traditional Chinese Medicine (10) leading in total publications, with the notable exception of the Canadian institution, University of Manitoba (which ranked 3rd overall) (Table 2). The University of Manitoba (Canada) and University of California, Los Angeles (USA) stood out for their high average citations (72.7 and 55.6, respectively), reflecting influential contributions despite moderate publication volumes (Figures 4A,B).

**Table 2:** Top 10 most productive institutions in IBD neuroimaging.

Rank	Institution	Documents	Citations	Average citations	total link strength	Country
1	Xidian University	14	392	28.0	43	China
2	Fudan University	12	336	28.0	43	China
3	University of Manitoba	11	800	72.7	26	Canada
4	Shanghai University of Traditional Chinese Medicine	10	373	37.3	36	China
5	Shanghai Jiao Tong University	9	280	31.1	37	China
6	University of Bologna	8	305	38.1	23	Italy
7	University of California, Los Angeles	8	445	55.6	24	USA
8	Heidelberg University	7	156	22.3	13	Germany
9	University of Modena and Reggio Emilia	7	231	33.0	21	Italy
10	Indiana University School of Medicine	5	233	46.6	24	USA

Author collaboration networks identified key research leaders. Liu Peng from Xidian University led in publication output (13 documents) and total link strength (124, Table 3), serving as a central hub for collaborative research. Bernstein Charles N. and Mayer Emeran A. demonstrated the highest average citations (72.7 and 56.2, respectively), reflecting their significant impact on the field. The network revealed distinct clusters: Liu’s team showed intensive, institution-based cohesion, while Bernstein and Mayer maintained sustained, influential international collaborations (Figures 4C,D).

**Table 3:** Top 10 most productive authors in IBD neuroimaging.

Rank	Author	Documents	Citations	Average citations	Total link strength	h_index	g_index	m_index	PY_start	Affiliated institution
1	Liu, Peng	13	390	30.0	124	10	13	0.833	2015	Xidian University
2	Bernstein, Charles N.	11	800	72.7	71	7	11	0.280	2002	University of Manitoba
3	Mayer, Emeran A.	10	562	56.2	81	9	10	0.409	2005	University of California, Los Angeles
4	Kornelsen, Jennifer	9	121	13.4	65	5	9	0.714	2020	University of Manitoba
5	Labus, Jennifer S.	9	529	58.8	69	8	8	0.615	2014	University of California, Los Angeles
6	Agostini, Alessandro	8	305	38.1	75	6	8	0.375	2011	University of Bologna
7	Bao, Chunhui	8	330	41.3	87	7	7	0.583	2015	Shanghai University of Traditional Chinese Medicine
8	Benuzzi, Francesca	7	231	33.0	64	5	7	0.357	2013	University of Modena and Reggio Emilia
9	Ebert, Matthias Philip	7	184	26.3	57	6	7	0.545	2016	Heidelberg University
10	Gionchetti, Paolo	7	254	36.3	65	5	7	0.313	2011	University

**Figure 5:**
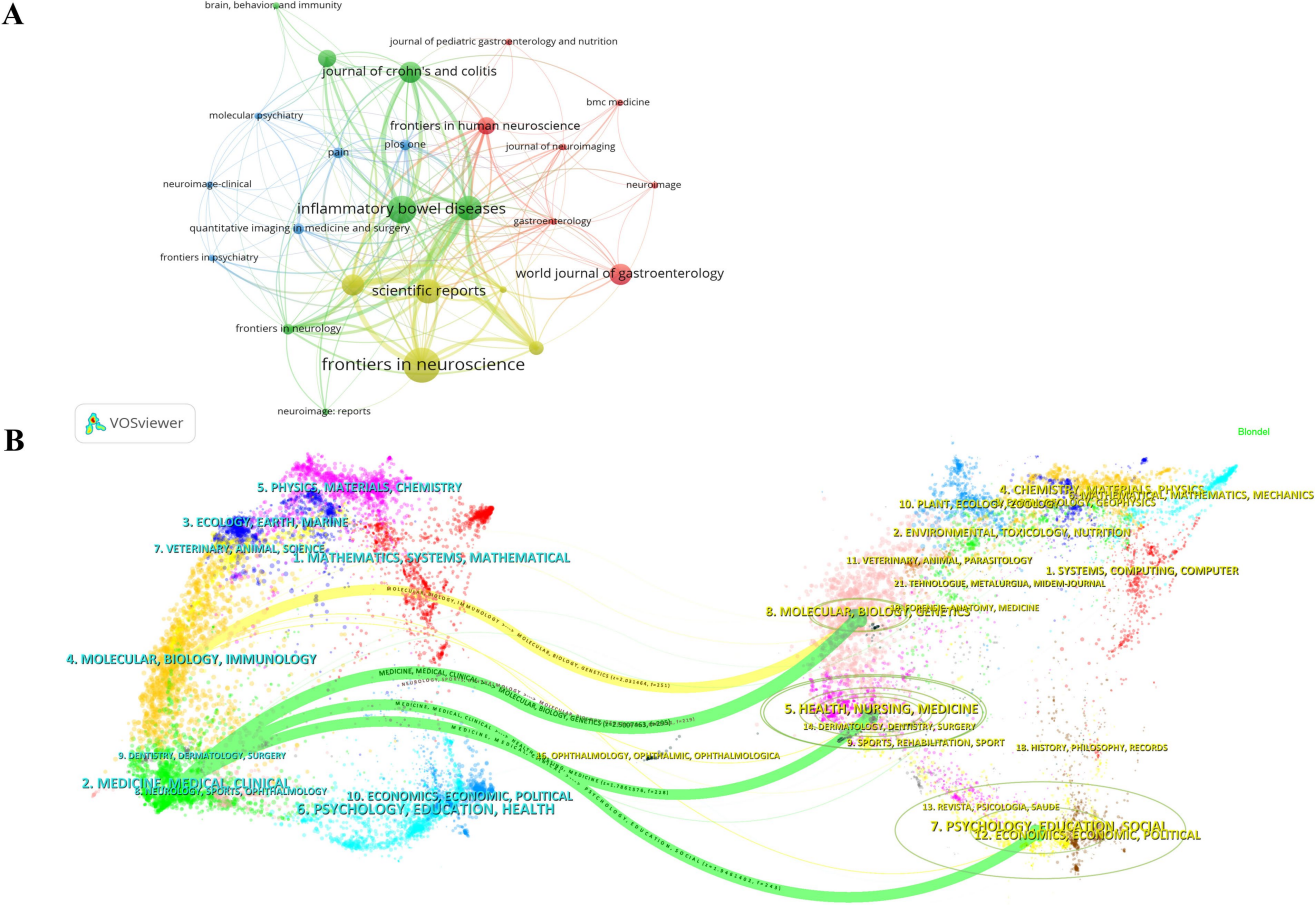
Visualization of journal citation networks and interdisciplinary knowledge flow in IBD neuroimaging research. **(A)** Journal citation network analysis using VOSviewer. The network was constructed with thresholds of minimum publications ≥ 2 and minimum total citations ≥ 20, yielding four distinct clusters. Node size is proportional to total citations, colors denote clusters, and lines indicate citation links between journals. **(B)** Journal dual-map overlay analysis using CiteSpace. The left and right panels display cited and citing journal clusters, respectively. Colored curves represent citation paths across disciplines, with thickness reflecting citation intensity, revealing cross-disciplinary knowledge transfer patterns.

Journal analysis identified core publishing platforms. *Frontiers in Neuroscience* led in publication output (10 documents), while *Neurogastroenterology and Motility* exhibited the highest citation impact (total citations: 505; average: 72.1, Table 4). The journal citation network revealed four thematic clusters, integrating gastroenterology with neuroimaging, brain-behavior-immune mechanisms, psychiatric comorbidities, and foundational neuroscience methodology (Figure 5A). The journal dual-map overlay visualizes the citation relationships between journals publishing inflammatory bowel disease (IBD) neuroimaging research (left cluster, citing journals) and the journals that are cited most frequently (right cluster, cited journals), with the z-scores (standardized association strength) and frequencies (f, number of citing relationships) annotated on the connecting paths, highlighting the field’s strong multidisciplinary integration (Figure 5B). The most prominent citation trajectories originate from Cluster 2 (Medicine, Medical, Clinical, including neurology, psychiatry, and gastroenterology journals), primarily targeting Cluster 5 (Health, Nursing, Medicine) (z = 1.78, *f* = 228), Cluster 8 (Molecular, Biology, Genetics) (z = 2.50, *f* = 295), and Cluster 7 (Psychology, Education, Social) (z = 1.94, *f* = 243). A second major trajectory originates from Cluster 4 (Molecular, Biology, Immunology) and also links to Cluster 8 (Molecular, Biology, Genetics) (z = 2.03, *f* = 251). These strong citation links reveal that IBD neuroimaging research is fundamentally built upon the translation of basic discoveries in molecular biology and immunology into clinical gastroenterology and neuroscience applications, underscoring the field’s strong multidisciplinary integration.

**Table 4:** Top 10 most productive journals in IBD neuroimaging.

Rank	Source	Documents	Citations	Average citations	Total link strength	h_index	g_index	m_index	PY_start	JCR quartile	2024 Impact factor
1	*Frontiers in Neuroscience*	10	48	4.8	93	5	6	1.000	2022	Q2	3.2
2	*Inflammatory Bowel Diseases*	8	166	20.8	95	5	8	0.313	2011	Q1	4.3
3	*Neurogastroenterology and Motility*	7	505	72.1	151	6	7	0.429	2013	Q1	2.9
4	*Scientific Reports*	7	160	22.9	89	5	7	0.455	2016	Q1	3.9
5	*Brain Imaging and Behavior*	6	140	23.3	133	5	6	0.556	2018	Q2	2.4
6	*Journal of Crohn’s and Colitis*	6	153	25.5	75	3	4	0.250	2015	Q1	8.7
7	*World Journal of Gastroenterology*	6	320	53.3	27	4	6	0.308	2014	Q1	5.4
8	*Frontiers in Human Neuroscience*	5	120	24.0	58	4	5	0.667	2021	Q2	2.7
9	*Frontiers in Immunology*	5	144	28.8	29	3	5	0.273	2016	Q1	5.9
10	*Brain and Behavior*	4	62	15.5	73	2	4	0.333	2021	Q2	2.7

#### 3.1.2 Knowledge base and research hotspots

**Figure 6:**
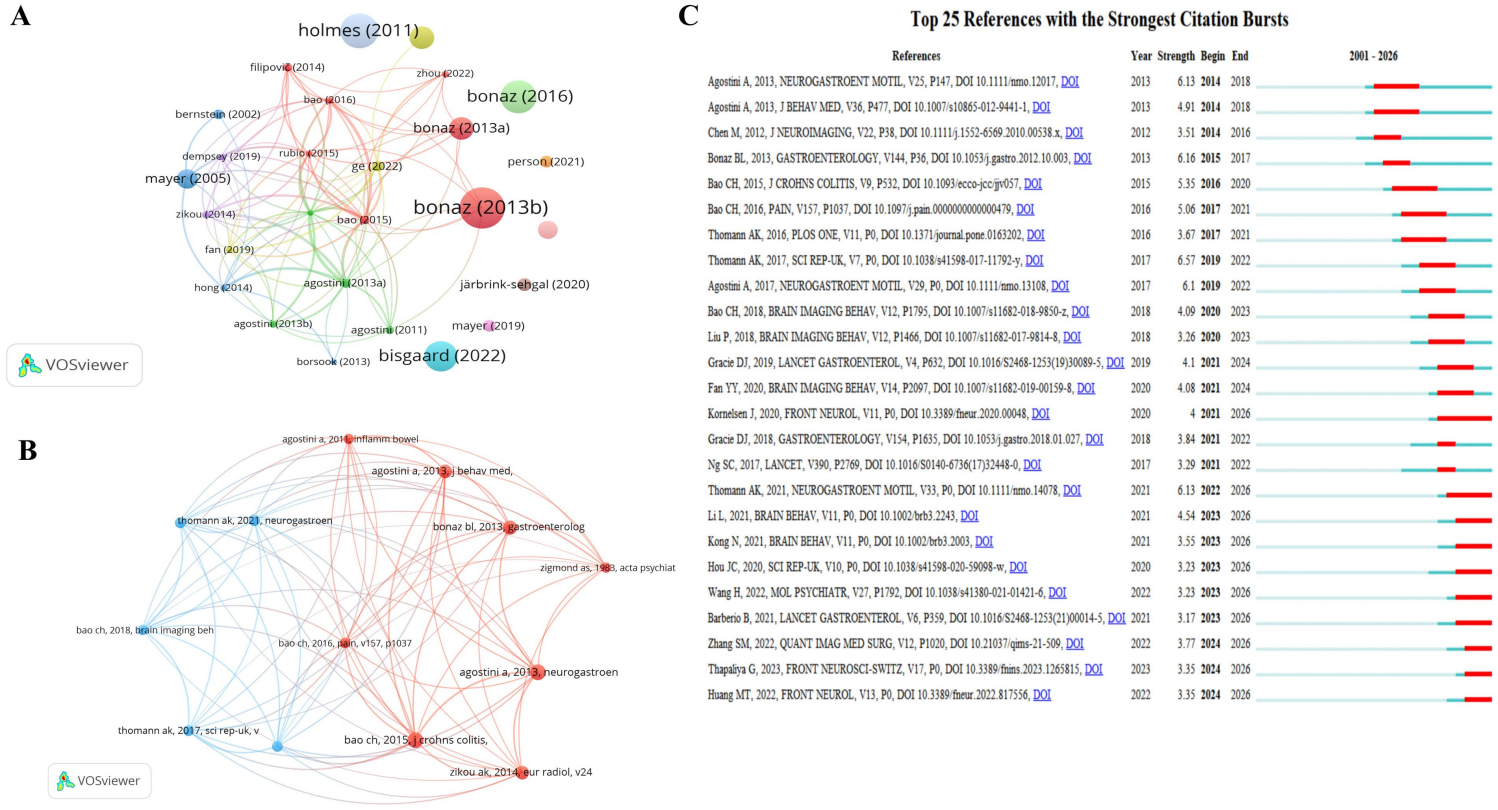
Reference network analysis and citation burst detection in IBD neuroimaging research. **(A)** Bibliographic coupling network of documents generated using VOSviewer (threshold: documents cited ≥ 50). Node size reflects citation count; lines denote coupling strength. **(B)** Co-citation network of references generated using VOSviewer (threshold: references co-cited ≥ 20). Node size reflects co-citation frequency; lines denote co-citation strength. **(C)** Top 25 references with the strongest citation bursts identified using CiteSpace. Red bars indicate burst duration and strength for each reference.

Citation analysis characterized the intellectual foundation of IBD neuroimaging research. The bibliographic coupling network identified Bonaz et al. (2013, Gastroenterology) as the most influential document (586 citations, Table 5), focusing on brain-gut interactions in IBD. Other core documents addressed gut microbiome-host signaling (Holmes et al., 2011, 466 citations), vagus nerve stimulation (Bonaz et al., 2013, 268 citations), and psychiatric comorbidities (Bisgaard et al., 2022, 396 citations), highlighting the brain-gut axis, neuroimmunomodulation, and psychological factors as foundational themes (Figure 6A). The co-citation network identified key theoretical and methodological underpinnings, with top co-cited references including Agostini et al. (2013, Neurogastroenterol)and Bao et al. (2015, J Crohns Colitis) (both 34 co-citations), which established voxel-based morphometry (VBM) application paradigms and abdominal pain-related brain abnormalities in IBD research (Figure 6B; Table 6). These foundational studies thus laid the groundwork for the subsequent recognition that structural brain alterations in IBD preferentially target an emotional and interoceptive network comprising the insula, ACC, and amygdala (see Section 3.2.2). Citation burst detection revealed dynamic evolution of research priorities: early influential bursts (2013–2018) were led by Agostini et al. (2013) (burst strength = 6.13), while recent bursts (2020–2026) centered on Li et al. (2021, Brain Behav) (4.54), Kong et al. (2021) (3.55), and Huang et al. (2022) (3.35), reflecting the evolving focus toward advanced neuroimaging techniques and emerging brain-gut interactions (Figure 6C).

**Table 5:** Top 10 most cited papers in IBD neuroimaging.

Rank	Paper	DOI	Total citations	TC per year	Normalized TC
1	Bonaz BL, 2013, GASTROENTEROLOGY	10.1053/j.gastro.2012.10.003	586	41.86	3.73
2	Holmes EC, 2011, TRENDS IN MICROBIOLOGY	10.1016/j.tim.2011.05.006	466	29.13	1.73
3	Bonaz BL, 2016, JOURNAL OF PHYSIOLOGY	10.1113/JP271539	436	39.64	6.33
4	Bisgaard TH, 2022, NATURE REVIEWS GASTROENTEROLOGY AND HEPATOLOGY	10.1038/s41575-022-00634-6	396	79.20	8.89
5	Bhatia VK, 2005, JOURNAL OF GASTROENTEROLOGY AND HEPATOLOGY (AUSTRALIA)	10.1111/j.1440-1746.2004.03508.x	271	12.32	1.10
6	Bonaz BL, 2013, NEUROGASTROENTEROLOGY AND MOTILITY	10.1111/nmo.12076	268	19.14	1.71
7	Mayer EA, 2005, PAIN	10.1016/j.pain.2005.03.023	220	10.00	0.90
8	He Z, 2017, WORLD JOURNAL OF GASTROENTEROLOGY	10.3748/wjg.v23.i19.3565	206	20.60	4.21
9	Järbrink-sehgal ME, 2020, CURRENT OPINION IN NEUROBIOLOGY	10.1016/j.conb.2020.01.016	138	19.71	4.68
10	Person H, 2021, PROGRESS IN NEURO-PSYCHOPHARMACOLOGY AND BIOLOGICAL PSYCHIATRY	10.1016/j.pnpbp.2020.110209	120	20.00	4.24

**Table 6:** Top 10 co-cited references in IBD neuroimaging.

Rank	Co-cited Reference	Citations	Total link strength
1	Agostini A, 2013, NEUROGASTROENTEROL MOTIL, V25, P147, DOI 10.1111/nmo.12017	34	313
2	Bao CH, 2015, J CROHNS COLITIS, V9, P532, DOI 10.1093/ecco-jcc/jjv057	34	366
3	Bonaz BL, 2013, GASTROENTEROLOGY, V144, P36, DOI 10.1053/j.gastro.2012.10.003	31	284
4	Agostini A, 2013, J BEHAV MED, V36, P477, DOI 10.1007/s10865-012-9441-1	29	309
5	Zikou AK, 2014, EUR RADIOL, V24, P2499, DOI 10.1007/s00330-014-3242-6	29	244
6	Thomann AK, 2021, NEUROGASTROENTEROL MOTIL, V33, DOI 10.1111/nmo.14078	25	299
7	Agostini A, 2017, NEUROGASTROENTEROL MOTIL, V29, DOI 10.1111/nmo.13108	24	318
8	Kornelsen J, 2020, FRONT NEUROL, V11, DOI 10.3389/fneur.2020.00048	24	298
9	Thomann AK, 2017, SCI REP-UK, V7, DOI 10.1038/s41598-017-11792-y	24	290
10	Agostini A, 2011, INFLAMM BOWEL DIS, V17, P1769, DOI 10.1002/ibd.21549	23	241

**Figure 7:**
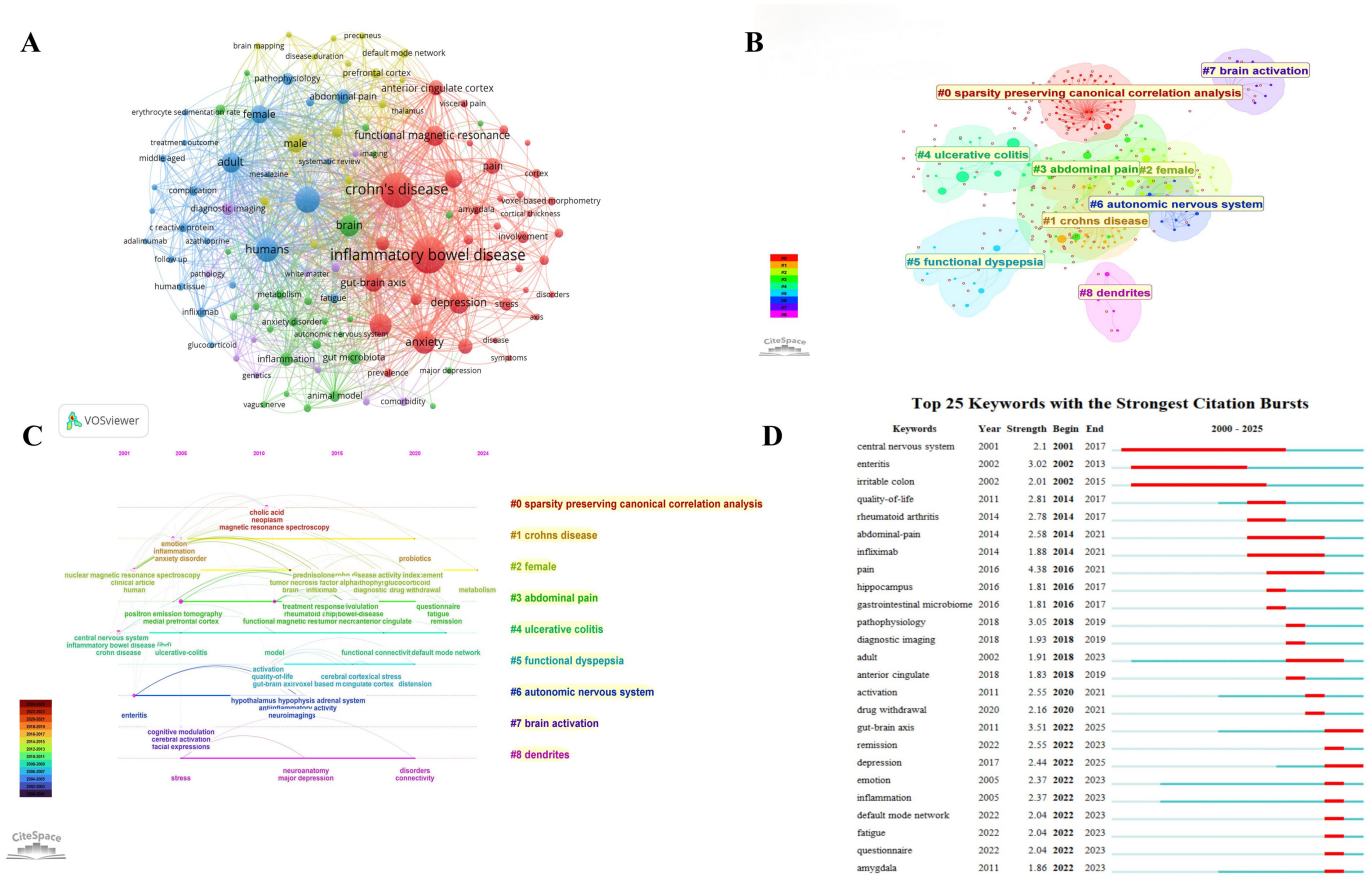
Keyword co-occurrence and evolutionary trends in IBD neuroimaging research. **(A)** Keyword co-occurrence network generated using VOSviewer (minimum occurrence threshold = 5, yielding five clusters). Node size is proportional to occurrence frequency, colors denote clusters, and lines indicate co-occurrence links. **(B)** Keyword clustering map generated using CiteSpace, visualizing thematic keyword clusters with colors denoting distinct research themes. **(C)** Keyword timeline visualization generated using CiteSpace, illustrating the temporal evolution of keyword clusters and dynamic shifts in research focus. **(D)** Top 25 keywords with the strongest citation bursts identified using CiteSpace, highlighting emerging research trends.

Keyword analysis delineated the thematic structure and temporal evolution of the field. The co-occurrence network revealed five major thematic clusters centered on core neuroanatomical regions (default mode network [DMN] prefrontal cortex anterior cingulate cortex [ACC]) specific disease conditions (CDUC) key research foci (abdominal pain anxiety depression) brain-gut interactions and autonomic nervous system function (Figure 7A). Cluster and timeline analyses further consolidated these themes into distinct research communities (Figure 7B). Keyword timeline analysis revealed dynamic shifts in research priorities: early work (pre-2010) centered on foundational IBD and neural correlates; the 2010s saw expanded neuroimaging applications (fMRI magnetic resonance spectroscopy [MRS]); recent years (2020–2025) shifted toward integrative models with growing focus on the gut-brain axis emotional regulation and DMN function (Figure 7C). Burst keyword analysis identified emerging hotspots with notable bursts including gut-brain axis (strength = 3.312022–2025) depression (2.442022–2023) inflammation (2.372022–2023) and DMN (2.042022–2023) underscoring growing interest in investigating bidirectional brain-gut interactions psychological comorbidities and network-level brain function in IBD (Figure 7D). Notably the centrality of the DMN and ACC in the keyword co-occurrence network (Figure 7A) mirrors their prominence as core altered networks in the neuroimaging literature.

#### 3.1.3 Clinical research progress

A total of seven clinical trials were retrieved from the PubMed database (Appendix 1). While clinical research in this field remains limited, these trials have outlined four thematic directions: (1) Neuroimaging and biomarker development, focusing on multimodal imaging strategies and biological markers for the objective assessment of IBD disease activity; (2) Clinical symptoms and neurobiological mechanisms, characterizing pain, sleep disturbances, and their underlying neural or receptor-related pathways in IBD; (3) Neuromodulation of brain function, examining the effects of non-invasive brain stimulation and complementary interventions on brain connectivity and functional responses in IBD patients; and (4) Psychological and behavioral interventions, evaluating mindfulness-based cognitive therapy and related approaches for alleviating psychological distress, depression, and sleep disturbances in this population.

### 3.2 Results of literature review

#### 3.2.1 Core neuroimaging modalities and technical applications in IBD

Structural neuroimaging serves as the foundational modality for investigating brain alterations in IBD. Utilizing T1/T2-weighted imaging and diffusion tensor imaging (DTI), this approach primarily focuses on detecting abnormalities in gray matter volume (GMV) and white matter microarchitecture. VBM studies have consistently reported reduced GMV in the insula, ACC, and thalamus in patients with IBD, with these alterations correlating with anxiety symptoms (Kornelsen et al., 2022; Kong et al., 2024; Zhang et al., 2025). A comprehensive surface-based morphometry study further reported left-lateralized cortical thinning and increased cortical complexity in IBD patients compared to healthy controls, suggesting that structural alterations extend beyond GMV reductions (Kornelsen et al., 2023). Complementary DTI studies have reported aberrant white matter diffusivity parameters in the hippocampal region and altered limbic system structural connectivity (Hou et al., 2020). The primary advantage of these techniques lies in their non-invasive nature and capacity to quantitatively capture organic structural changes; as inherently structural techniques, they do not assess functional brain activity (Yin et al., 2026).

Functional neuroimaging techniques, which capture real-time brain activity and neural network interactions, have been widely adopted in IBD research. Resting-state functional magnetic resonance imaging (rs-fMRI) represents the most extensively applied modality in this field. Rs-fMRI investigations employ metrics such as amplitude of low-frequency fluctuation (ALFF), regional homogeneity (ReHo), and functional connectivity (FC) to characterize spontaneous brain activity and network connectivity patterns in patients with IBD under resting conditions (Thomann et al., 2021; Thapaliya et al., 2023b; Huang et al., 2024). At the level of regional brain activity, several studies have reported increased ALFF and ReHo values in the ACC and superior frontal gyrus in patients with CD, with these alterations being positively correlated with anxiety scores and fatigue severity (Kong et al., 2021; Kong et al., 2024). In contrast, patients with UC exhibit predominantly reduced ALFF in the hippocampus and parahippocampal gyrus, which is associated with working memory deficits (Fan et al., 2019). At the network level, rs-fMRI studies generally report aberrant FC within the DMN, salience network (SN), and executive control network (ECN) in patients with IBD (Alyami, 2025; Yin et al., 2026). Task-based fMRI employs specific stimulus paradigms to elucidate brain activation patterns and has utilized three main paradigm types: pain stimulation, emotional provocation, and cognitive tasks (Rubio et al., 2016; Nair et al., 2019; Öhlmann et al., 2023; Guo et al., 2024; Lanters et al., 2024). Pain stimulation paradigms have revealed reduced ACC activation and enhanced deactivation of somatosensory cortices during rectal distension in patients with IBD, distinctly differentiating them from healthy controls and individuals with irritable bowel syndrome (Rubio et al., 2016). Emotional provocation paradigms have revealed aberrant activation patterns in the amygdala and prefrontal cortex when patients with IBD are exposed to negative emotional stimuli, with the degree of abnormality correlating with anxiety and depression severity (Lanters et al., 2024). Cognitive task paradigms have shown that patients with CD in remission exhibit enhanced bilateral hemisphere activation during verbal fluency tasks, with younger patients displaying activation patterns resembling those of healthy older controls, suggesting accelerated cognitive-related brain aging (Nair et al., 2019). Electroencephalography (EEG) studies in IBD remain relatively limited. One study reported that intermittent generalized slow waves in the theta band represent the most common EEG abnormality in patients with CD (Kelleci et al., 2019). Leveraging its high temporal resolution, EEG has shown potential in monitoring therapeutic response to vagus nerve stimulation, as changes in EEG power spectra reflect treatment-induced modulation of central neural activity (Kibleur et al., 2018).

Molecular neuroimaging techniques, which focus on detecting molecular-level alterations within the brain, primarily include proton magnetic resonance spectroscopy (^1^H-MRS) and positron emission tomography (PET). ^1^H-MRS studies have revealed elevated glutamate-related ratios and reduced gamma-aminobutyric acid (GABA) ratios in the bilateral ACC of patients with CD experiencing abdominal pain, with these metabolic changes positively correlated with pain intensity (Lv et al., 2018). Ultra-high-field 7 T MRS studies have further demonstrated associations between insular metabolite levels and gastrointestinal symptoms as well as pain catastrophizing tendencies (Berthold et al., 2025). PET investigations remain largely confined to animal models, with a primary focus on detecting neuroinflammation (Kurtys et al., 2017; Largeau et al., 2017); clinical translation of these approaches warrants further exploration.

Multimodal imaging integration has emerged as a research hotspot, encompassing structural–functional MRI fusion and combined imaging–clinical parameter analyses (Yin et al., 2026). Structural–functional integration studies have reported concurrent GMV reduction, FC abnormalities, and neurotransmitter metabolic alterations in key brain regions such as the ACC and hippocampus in patients with CD (Wang et al., 2023). Regarding imaging–clinical parameter integration, diffusion kurtosis imaging and intravoxel incoherent motion (IVIM) techniques have shown promise in effectively differentiating CD patients with and without anxiety based on cerebral microstructural and perfusion abnormalities (Yue et al., 2025). The primary advantage of these multimodal approaches lies in their capacity to probe brain-gut axis pathways from multiple dimensions; however, their widespread adoption is hindered by complex post-processing requirements and the absence of standardized protocols. Collectively, the technical evolution from unimodal structural descriptions to multimodal integration reflects a maturing field. However, the marked heterogeneity in acquisition parameters, preprocessing pipelines, and analytical frameworks across studies remains a critical barrier to cross-study comparability and meta-analytic synthesis, and few studies have evaluated whether multimodal approaches yield incremental value over unimodal methods. This evolution, summarized in Table 7, underscores a growing recognition that IBD-related brain alterations are multidimensional and require comprehensive phenotyping.

**Table 7:** Studies related to different neuroimaging modalities of IBD.

Imaging modality	Study paradigm	Analytical indicators	IBD Common features	Key findings in UC	Key findings in CD
Structural neuroimaging	sMRI	VBM, cortical thickness, surface area, gyrus gyrification	Abnormal GMV and cortical thickness are observed in emotion/pain-related brain regions, including the insula, ACC, mPFC, and limbic system (amygdala, thalamus, hippocampus)	Decreased GMV in the insula, thalamus, and other limbic system regions; increased GMV in the putamen and SMA, with more significant changes in the active phase (Zhang et al., 2022)	Decreased GMV in the insula and ACC; decreased GMV in the medial frontal lobe; reduced GMV/WMV and cortical thickness in the left precentral gyrus and frontal regions (Bao et al., 2017; Thapaliya et al., 2023a; Kong et al., 2024); left-lateralized cortical thinning and increased cortical complexity in IBD, suggesting that structural alterations extend beyond GMV reductions (Kornelsen et al., 2023)
	DTI	MD, AD, RD, TBSS	Microstructural abnormalities are present in multiple white matter tracts, including the corticospinal tract, corona radiata, and corpus callosum; aberrant diffusivity parameters in the hippocampal region have been associated with disrupted limbic system connectivity; changes in diffusion indicators are associated with clinical symptoms such as pain and anxiety	Limited relevant studies; mainly manifested by decreased diffusion indicators in white matter regions such as the corticospinal tract and corona radiata (Zhang et al., 2025)	Microstructural abnormalities in language-related white matter tracts; changes in diffusion indicators in some white matter regions are associated with anxiety and disease duration (Hou et al., 2020; Yue et al., 2025)
	DKI	Kurtosis, ADC, perfusion-related indicators	Abnormal diffusion kurtosis and perfusion indicators in brain regions such as the right insula, left insula, and right thalamus, which are associated with emotional symptoms such as anxiety	Limited relevant studies; no clear specific changes were found	In active CD patients with anxiety, increased ADCslow in the left insula and decreased kurtosis in the right insula were observed (Yue et al., 2025)
Functional Neuroimaging	rs-fMRI	ReHo, ALFF, DC, FC, ICA, graph theory analysis	Dysfunctional connectivity in networks such as the DMN, SN, ECN, and limbic system (amygdala, ACC); abnormal ReHo/ALFF in pain- and emotion-related brain regions including the insula, MCC, and ACC	Increased intranetwork connectivity in the auditory network; abnormal internetwork connectivity between the dorsal attention network and DMN, which is associated with anxiety and depression (Ren et al., 2025); decreased ALFF in the hippocampus and parahippocampal gyrus, associated with working memory deficits (Fan et al., 2019); inability to distinguish visceral pain from somatic pain in the quiescent phase (Öhlmann et al., 2023)	Decreased ReHo in the insula and MCC in patients with pain; decreased FC between the PAG and DMN (Bao et al., 2016; Chen et al., 2023); abnormal FC in the DMN and ECN, which is associated with anxiety and cognition (Thomann et al., 2017; Liu et al., 2018); weaker association between executive function and cerebello-cortical FC with visual processing areas (Kornelsen et al., 2025); reduced FC between the superior parietal lobule and parahippocampal gyrus/hippocampus associated with fatigue, moderated by disease activity (McIver et al., 2025)
	Task-based fMRI	BOLD signal analysis, pain intensity matching, activation and FC analysis	Abnormal activation of pain matrix-related brain regions such as the insula, ACC, and somatosensory cortex under pain stimulation; abnormal function of brain regions related to visceral and somatic pain processing (interoceptive and perceptual brain regions)	Decreased activation of interoceptive brain regions under rectal distension stimulation; inability to distinguish visceral pain from somatic pain at the neural and behavioral levels (Mayer et al., 2005; Öhlmann et al., 2023)	Increased activation of perceptual brain regions during pain experience; abnormal activation of brain regions related to pain anticipation (Huang et al., 2016; Yeung, 2021)
	EEG	Microstate analysis, subnetwork analysis, spatiotemporal brain state analysis	Altered brain state dynamics; abnormal electrical activity in emotion- and cognition-related brain regions such as the mPFC and insula	Increased global FC, decreased modularity, and the stability of the mPFC is associated with emotional symptoms (Wang et al., 2022)	Shifted brain state dynamics; enhanced connectivity between the insula and mPFC, which is associated with disease duration (Hall et al., 2023)
Molecular Neuroimaging	MRS	GABA+/Glx, Glu/tCr, Glx/tCr, Asp/NAAG, mIns	Abnormal metabolites in emotion- and pain-related brain regions such as the ACC and left insula, which are associated with clinical symptoms including pain, anxiety, and depression	Limited relevant studies; no clear specific metabolic changes were found	Increased Glu/tCr and Glx/tCr, and decreased GABA+/tCr in the ACC, which are associated with pain and anxiety (Lv et al., 2018); metabolites in the left insula are associated with pain catastrophizing (Berthold et al., 2025)
	PET	TSPO expression, NK-1R BP, rCBF	Increased TSPO expression in neuroinflammation-related brain regions such as the insula and ACC; decreased NK-1R BP in extensive cortical and subcortical regions (limbic system, frontal lobe)	Under rectal distension stimulation, activation of the limbic system and frontal lobe is weaker than that in IBS, with endogenous pain inhibition (Mayer et al., 2005)	Increased TSPO expression in neuroinflammation-related brain regions; decreased NK-1R BP in extensive regions, which is associated with clinical symptoms (Jarcho et al., 2013; Huang et al., 2016)
	GluCEST MRI	GluCEST value	Decreased GluCEST value in the left dId, which is closely associated with depressive symptoms	Limited relevant studies; no clear specific changes were found	Decreased GluCEST value in the left dId in remissive CD patients, which is positively correlated with depression scores (Xu et al., 2025)
Multimodal Fusion	sMRI+rs-fMRI, sMRI+rs-fMRI+MRS, sMRI+DTI, etc.	Combined multi-dimensional indicators including structure, function, and metabolism; mediation analysis, joint analysis	Multi-dimensional brain abnormalities (such as those in the insula, ACC, and mPFC) are interrelated, jointly participating in the abnormal regulation of the brain-gut axis in IBD; it can more comprehensively reflect disease-related brain changes	Limited relevant studies; mainly manifested by the synergy of structural and functional abnormalities, which are associated with emotional and cognitive symptoms (Wang et al., 2022)	Abnormal GABA+/Glx metabolism in the mPFC mediates the severity of depression; the synergy of structural and functional abnormalities is associated with pain, anxiety, and cognition (Wang et al., 2023; Kong et al., 2024)

ACC, anterior cingulate cortex; ADC, apparent diffusion coefficient; ADCslow, slow diffusion coefficient; AD, axial diffusivity; ALFF, amplitude of low-frequency fluctuation; BOLD, blood oxygen level dependent; BP, binding potential; CD, Crohn’s disease; DC, degree centrality; dId, dorsal dysgranular insula; DKI, diffusion kurtosis imaging; DMN, default mode network; DTI, diffusion tensor imaging; ECN, executive control network; EEG, electroencephalography; FC, functional connectivity; GABA, gamma-aminobutyric acid; GluCEST MRI, glutamate chemical exchange saturation transfer MRI; GMV, gray matter volume; IBD, inflammatory bowel disease; IBS, irritable bowel syndrome; ICA, independent component analysis; IVIM, intravoxel incoherent motion imaging; MCC, middle cingulate cortex; MD, mean diffusivity; mPFC, medial prefrontal cortex; MRS, Magnetic resonance spectroscopy; NK-1R, Neurokinin-1 Receptor; PAG, periaqueductal gray; PET, positron emission tomography; rCBF, regional cerebral blood flow; RD, radial diffusivity; ReHo, regional homogeneity; rs-fMRI, resting-state functional magnetic resonance imaging; SMA, supplementary motor Area; sMRI, structural magnetic resonance imaging; SN, salience network; TBSS, tract-based spatial statistics; TSPO, translocator protein; UC, ulcerative colitis; VBM, voxel-based morphometry; WMV, white matter volume.

#### 3.2.2 Key brain regions and networks: altered patterns and clinical correlates

Patients with IBD frequently present with systemic symptoms including depression, anxiety, chronic abdominal pain, severe fatigue, and cognitive impairment. These manifestations are closely associated with structural and functional abnormalities in specific brain regions and networks, with certain alterations persisting independently of intestinal inflammation severity, representing core neuroimaging correlates of brain-gut axis dysfunction (Ge et al., 2022). A summary of neuroimaging features related to different symptoms in IBD is presented in Table 8.

**Table 8:** Neuroimaging features related to different symptoms in IBD.

Symptoms	Main Brain regions/associated networks	Neuroimaging features - IBD commonality	Neuroimaging features - CD specificity	Neuroimaging features - UC specificity	Representative studies
Anxiety and Depression	Amygdala, ACC, mPFC, DMN, limbic system, auditory network, dorsal attention network	Abnormal FC of emotion regulation networks; abnormal FC related to the amygdala; abnormal GABA+/Glx metabolism in the mPFC; changes in GMV/cortical thickness in relevant brain regions (Thomann et al., 2017; Liu et al., 2018; Fan et al., 2020; Kong et al., 2024; Ren et al., 2025; Yang et al., 2025)	Increased FC within the DMN in the remission phase; FC strength of the MCC is positively correlated with anxiety scores; abnormal local topological structure of the ACC and mPFC in patients with emotional disorders (Kong et al., 2021; Kong et al., 2024)	Enhanced intranetwork connectivity of the auditory network; abnormal internetwork connectivity between the dorsal attention network and DMN, associated with emotional symptoms (Ren et al., 2025).	Thomann et al. (2017), Liu et al. (2018), Fan et al. (2020), Kong et al. (2024), Ren et al. (2025), Yang et al. (2025)
Abdominal Pain	Insula, ACC, MCC, DMN, SN, somatosensory cortex, pain matrix	Abnormal function of pain matrix-related brain regions; visceral hypersensitivity associated with abnormal pain matrix activation; decreased GMV and disordered FC in pain-related brain regions (Huang et al., 2016; Bao et al., 2017; Chen et al., 2023; Öhlmann et al., 2023; Wu et al., 2025)	Decreased GMV in the insula and ACC in patients with pain (Bao et al., 2015; Bao et al., 2017); decreased ReHo in the insula and MCC (Bao et al., 2016); decreased FC between the PAG and DMN, increased FC between the ACC and DMN, correlated with pain scores(Chen et al., 2023; Wu et al., 2025)	Inability to distinguish visceral pain from somatic pain at neural and behavioral levels in the quiescent phase (Öhlmann et al., 2023); visceral pain threshold associated with gastrointestinal symptoms and chronic stress (Takahashi et al., 2021; Öhlmann et al., 2023)	Huang et al. (2016), Bao et al. (2017), Chen et al. (2023), Öhlmann et al. (2023), Wu et al. (2025)
Fatigue	SMA, left precentral gyrus, frontal regions, cerebellum, limbic system, DMN	Decreased GMV/WMV/cortical thickness in sensorimotor and frontal regions (Thomann et al., 2016; Thapaliya et al., 2023a; Thomann et al., 2025); abnormal FC of brain networks associated with fatigue severity (McIver et al., 2025)	Atrophy of the SMA in the active phase, associated with fatigue severity (Thapaliya et al., 2023a; Thomann et al., 2024); decreased GMV/WMV and cortical thickness in the left precentral gyrus and frontal regions (Thomann et al., 2016; Thapaliya et al., 2023a; Thomann et al., 2025)	Limited relevant studies; no clear specific imaging features identified	Thomann et al. (2016), Thomann et al. (2022), Thapaliya et al. (2023a), Thomann et al. (2024), Yang et al. (2024a), Mciver et al. (2025), Thomann et al. (2025)
Cognitive Impairment	Prefrontal cortex, medial frontal cortex, paracentral lobule, cingulate gyrus, DMN, ECN, cerebellum, hippocampus	Decreased GMV and abnormal white matter microstructure in cognition-related brain regions; disordered FC of DMN and ECN (Sun et al., 2022; Patel et al., 2023; Guo et al., 2024; Li F. et al., 2024; Bao et al., 2026); abnormal language-related white matter tracts (Nair et al., 2019)	Persistent abnormal bilateral hemisphere activation related to cognition in the remission phas; accelerated brain aging; disordered DMN connectivity predicting cognitive impairment progression(Nair et al., 2019); weaker cerebello-cortical functional connectivity associated with poorer executive function (Kornelsen et al., 2025)	Bilateral hippocampal ALFF reduction and enhanced hippocampal–middle frontal gyrus FC in active UC, correlating with cognitive impairment severity(Fan et al., 2019)	Papathanasiou et al. (2014), Fan et al. (2019), Nair et al. (2019), Sun et al. (2022), Patel et al. (2023), Guo et al. (2024), Li F. et al. (2024), Christodoulou et al. (2025), Bao et al. (2026), Kornelsen et al. (2025)

ACC, anterior cingulate cortex; ALFF, amplitude of low-frequency fluctuation; CD, Crohn’s disease; DMN, default mode network; DTI-ALPS index, diffusion tensor image-analysis along the perivascular space index; ECN, executive control network; FC, functional connectivity; GABA, gamma-aminobutyric acid; GMV, gray matter volume; IBD, inflammatory bowel disease; MCC, middle cingulate cortex; mPFC, medial prefrontal cortex; PAG, periaqueductal gray; ReHo, regional homogeneity; SMA, supplementary motor area; SN, salience network; UC, ulcerative colitis; WMV, white matter volume.

Anxiety and depression are common psychiatric comorbidities in IBD (Bisgaard et al., 2022). Neuroimaging studies have demonstrated that these symptoms are associated with structural and functional abnormalities in core emotional regulation regions—including the ACC, amygdala, and ventromedial prefrontal cortex (vmPFC)—and with aberrant FC within the DMN, limbic system, and ECN as key correlates of anxiety and depression in patients with IBD (Thomann et al., 2017; Fan et al., 2020; Wang et al., 2023; Kong et al., 2024; Yang et al., 2025; Yin et al., 2026). Patients with CD and comorbid anxiety/depression exhibit disrupted local topological properties in the ACC and medial prefrontal cortex (mPFC) (Kong et al., 2021; Kong et al., 2024). Notably, some studies report that patients with CD in remission continue to exhibit emotional network abnormalities despite absent overt intestinal inflammation (Thomann et al., 2025), raising the possibility that certain brain alterations represent trait-like phenomena that are at least partially independent of peripheral inflammatory activity (Ge et al., 2022).

Chronic abdominal pain—a core symptom of IBD—is linked to structural and functional abnormalities within the pain matrix (insula, ACC, and somatosensory cortex), extending beyond the emotional regulation abnormalities described above (Bao et al., 2016; Yeung, 2021; Wu et al., 2025). Specifically, patients with CD and pain exhibit reduced GMV in the insula and ACC and decreased insular ReHo, with these measures negatively correlating with pain severity (Bao et al., 2015; Bao et al., 2017). FC abnormalities predominantly involve the DMN and SN, characterized by reduced periaqueductal gray (PAG)–DMN connectivity and increased ACC–DMN connectivity in pain-experiencing CD patients, both correlating with pain severity (Chen et al., 2023; Wu et al., 2025). Meta-analytic evidence supports reduced cingulate FC and decreased medial frontal GMV in CD, associated with pain anticipation and emotional processing (Yeung, 2021). Furthermore, transcranial direct current stimulation (tDCS) has shown preliminary efficacy in ameliorating chronic pain in IBD through modulation of FC in pain-related brain regions (Neeb et al., 2019).

Severe fatigue —an under-recognized symptom in IBD—is associated with functional abnormalities in the thalamus, basal ganglia, and DMN (Thomann et al., 2022; Thomann et al., 2024; Yang et al., 2024a; McIver et al., 2025; Thomann et al., 2025). Studies have reported that reduced GMV in sensorimotor regions correlates with fatigue symptoms in patients with CD, with this association being more pronounced in patients during remission, suggesting disease state-dependent neural substrates for fatigue (Thomann et al., 2025). rs-fMRI investigations have revealed aberrant DMN FC in CD patients, potentially contributing to severe fatigue through effects on energy metabolism and cognitive resource allocation (McIver et al., 2025). These regional brain abnormalities persist following resolution of intestinal inflammation (Thomann et al., 2025), suggesting that central contributions to fatigue may be at least partially dissociable from peripheral inflammatory activity.

Cognitive impairment in IBD is associated with structural and functional alterations in the hippocampus and prefrontal cortex. Patients with IBD frequently exhibit mild cognitive impairment, with neural substrates localized to the hippocampus, prefrontal cortex, and limbic system (Papathanasiou et al., 2014; Fan et al., 2019; Nair et al., 2019; Christodoulou et al., 2025; Bao et al., 2026). Structurally, reduced GMV in the hippocampus and prefrontal cortex has been reported in IBD patients, with some studies finding negative correlations with cognitive performance, though the strength and specificity of these associations vary (Sun et al., 2022; Patel et al., 2023; Guo et al., 2024; Li F. et al., 2024). Functionally, decreased ECN connectivity shows positive correlation with the degree of cognitive impairment (Guo et al., 2024; Li F. et al., 2024). Kornelsen et al. (2025) further reported that IBD patients show a weaker association between executive function and cerebello-cortical functional connectivity with visual processing areas compared to healthy controls, suggesting that cerebello-cortical dysconnectivity may contribute to cognitive deficits in IBD. Specifically, patients with active UC exhibit bilateral hippocampal ALFF reduction and enhanced hippocampal–middle frontal gyrus FC, correlating with cognitive impairment severity, implicating the limbic system as a core mediator of UC-related cognitive dysfunction via brain-gut axis mechanisms (Fan et al., 2019). Patients with UC additionally exhibit glymphatic system dysfunction, reflected by reduced DTI-ALPS index, which may serve as a sensitive imaging biomarker for cognitive impairment (Bao et al., 2026). Patients with CD in remission demonstrate enhanced bilateral hemispheric activation during language tasks, suggesting accelerated cognitive-related brain aging (Nair et al., 2019).

In summary, functional abnormalities within the anterior cingulate–insula–amygdala circuitry consistently correlate with abdominal pain intensity and anxiety/depression scores, while disrupted DMN connectivity is associated with fatigue and cognitive decline (see Table 8 for a detailed synthesis). Despite this convergence, the literature is characterized by considerable variability in effect sizes and spatial localization of findings, likely attributable to differences in sample composition, disease activity states, and analytical methodologies. Furthermore, whether these network-level alterations represent cause, consequence, or epiphenomenon of IBD-related brain-gut dysregulation remains unresolved, as most studies employ cross-sectional designs.

#### 3.2.3 Divergent and convergent neuroimaging features of CD and UC

As the two major subtypes of IBD, CD and UC exhibit both shared core abnormalities and distinguishable neuroimaging phenotypic profiles (Christodoulou et al., 2025). Rs-fMRI studies have generally reported structural and functional abnormalities in the DMN, SN, and limbic system (including the ACC, insula, and amygdala) in both CD and UC (Thomann et al., 2017; Kornelsen et al., 2022; Christodoulou et al., 2025). The insula–ACC–limbic pathway is considered a common vulnerable target of brain-gut axis dysfunction in IBD, closely associated with abdominal pain, anxiety, depression, and impaired interoceptive processing (Fan et al., 2020; Yang et al., 2024a). Additionally, cerebellar network connectivity alterations have been observed in both subtypes, pointing to a possible role of the cerebellum in emotional and cognitive regulation (Kornelsen et al., 2021; Li Y. F. et al., 2024).

Specifically, CD is primarily characterized by hyperactivity in anterior cognitive-affective networks, with increased ALFF and ReHo in the ACC and prefrontal cortex that correlate positively with anxiety and fatigue severity (Thomann et al., 2017; Hou et al., 2020; Kong et al., 2021; Kong et al., 2022; Sun et al., 2022). The DMN in CD exhibits state-dependent remodeling, characterized by enhanced connectivity during remission and reduced connectivity during active disease (Hou et al., 2020; Agostini et al., 2023). Alterations in language network and frontoparietal network connectivity are also prominent (Li et al., 2022). In contrast, UC is predominantly characterized by reduced activity in posterior memory-related regions, with significantly decreased ALFF in the hippocampus and parahippocampal gyrus that correlates with working memory deficits (Fan et al., 2019). Reduced connectivity within visual and cerebellar networks suggests visceral hypersensitivity and impaired sensorimotor integration (Kornelsen et al., 2021; Öhlmann et al., 2023). Collectively, CD preferentially involves higher-order cognitive networks, whereas UC predominantly affects sensory–memory network (Christodoulou et al., 2025). Emerging evidence suggests that these distinct phenotypic patterns may arise from different neuroinflammatory targets, which could lead to selective regional vulnerability.

Disease activity differentially modulates neuroimaging alterations in CD and UC. In patients with active CD, further increases in ACC ALFF and reduced SN connectivity have been observed (Thapaliya et al., 2023b; Thomann et al., 2024). During remission, heightened prefrontal activity and enhanced intra-DMN connectivity persist (Hou et al., 2019; Nair et al., 2019; Hou et al., 2020), suggesting that certain alterations may represent trait-like markers. Furthermore, a moderation analysis identified a significant interaction between disease activity, fatigue, and functional connectivity of the superior parietal lobule and parahippocampal gyrus/hippocampus in CD, indicating that disease activity modulates the relationship between brain connectivity and fatigue severity (McIver et al., 2025). In patients with active UC, hippocampal activity is markedly reduced, accompanied by compensatory increases in posterior cingulate cortex (PCC) activity (Fan et al., 2019; Bao et al., 2026). Patients with UC in remission exhibit impaired ability to differentiate visceral from somatic pain, with visceral pain thresholds correlating with gastrointestinal symptoms (Takahashi et al., 2021; Öhlmann et al., 2023), suggesting that prior intestinal inflammation may be associated with persistent central reorganization.

In summary, although head-to-head comparisons remain limited and UC studies are substantially fewer than CD studies, the two subtypes display distinguishable neuroimaging phenotypic profiles: CD is characterized by anterior cognitive-affective network hyperactivity (associated with anxiety, fatigue, and impaired cognitive control), whereas UC is characterized by posterior memory-related region hypoactivity (associated with visual–cerebellar network disruption and visceral hypersensitivity) (Christodoulou et al., 2025). This asymmetry in the evidence base limits the confidence with which subtype-specific profiles can be delineated, and the apparent phenotypic distinctions described above should be regarded as preliminary patterns requiring validation in larger, balanced cohorts. Both subtypes share abnormalities in the insula–ACC–limbic pathway, implicating this circuit as a vulnerable hub of brain-gut axis dysfunction in IBD (Christodoulou et al., 2025).

#### 3.2.4 Neuroimaging correlates of brain-gut axis pathways in IBD

The microbiota-gut-brain axis is hypothesized to serve as a critical regulatory pathway in IBD, potentially modulating central nervous system structure and function through microbial metabolites (e.g., short-chain fatty acids) and immune activation (Person and Keefer, 2021). Cross-sectional neuroimaging studies have shown that IBD patients with gut microbiota dysbiosis exhibit more pronounced abnormalities in the insula, ACC, and DMN—alterations closely associated with emotional disturbances and visceral hypersensitivity (Largeau et al., 2017; Person and Keefer, 2021; Qiu et al., 2022; Thomann et al., 2022). However, it is important to note that these studies primarily establish pairwise associations between microbiota profiles and brain metrics, without simultaneously assessing immune mediators presumed to serve as intermediate links along the microbiota-gut-brain axis. For instance, Thomann et al. reported correlations between microbial taxa abundance and depression/fatigue symptoms but did not test whether inflammatory markers formally mediate the relationship between microbiota and neuroimaging phenotypes (Thomann et al., 2022). Consequently, the mechanistic assumption that gut dysbiosis influences brain function through immune activation remains largely untested within a unified analytical framework in the current IBD neuroimaging literature.

The neuroimmune pathway is proposed as another route for gut-to-brain signaling in IBD. Peripheral inflammatory cytokines (e.g., tumor necrosis factor-*α* (TNF-α), interleukin-6 (IL-6)) can enter the central nervous system via the circulatory system (Dantzer, 2018), with the potential to trigger neuroinflammation and microglial activation. Neuroimaging studies have reported elevated translocator protein (TSPO) expression in brain regions associated with neuroinflammation in patients with IBD, accompanied by marked functional connectivity abnormalities within the salience network (Thomann et al., 2017). These neuroinflammatory changes show positive correlations with peripheral inflammatory levels (Kurtys et al., 2017; Largeau et al., 2017; Ge et al., 2022; He et al., 2025).

The vagal pathway represents an essential anatomical substrate for bidirectional brain-gut communication, conveying intestinal inflammatory signals to the central nervous system while simultaneously activating the cholinergic anti-inflammatory pathway to exert counter-regulatory effects on intestinal inflammation (Bonaz et al., 2013). Patients with IBD demonstrate FC abnormalities in vagal-associated brain regions, including the brainstem and ACC. Notably, improvements in vagal function following neuromodulatory interventions are accompanied by amelioration of both brain functional abnormalities and clinical symptoms (Bonaz et al., 2016; Brenner et al., 2021; D'Haens et al., 2023). However, studies investigating the synergistic interactions among these three pathways remain scarce, and notably, no study to date has simultaneously integrated gut microbiome profiling, peripheral immune markers, and multimodal neuroimaging within a unified analytical framework in an IBD cohort. This represents a significant barrier to disentangling the relative contributions of each pathway and their potential synergistic or compensatory interactions. Consequently, while the microbiota-gut-brain axis provides a compelling heuristic for organizing the existing evidence, its status as an empirically validated mechanism in IBD neuroimaging research remains aspirational. Future studies adopting multi-omics approaches that simultaneously profile the gut microbiome, peripheral immune markers, and brain structure/function with formal mediation or moderation analyses will be essential to move the field from parallel description to genuine integration.

#### 3.2.5 Progress toward clinical translation: current evidence and barriers

Neuroimaging holds considerable exploratory potential for informing clinical applications in IBD, including diagnosis, disease activity assessment, prognosis prediction, and treatment monitoring (Silva et al., 2024; Yin et al., 2026). However, it must be emphasized that this potential remains largely at the proof-of-concept stage, and none of the neuroimaging markers described below have yet been validated for routine clinical use. Regarding diagnostic utility, preliminary studies suggest that group-level neuroimaging differences can distinguish patients with IBD from healthy controls based on structural abnormalities in regions such as the insula and ACC, as well as associated network-level functional alterations, though these findings have not been evaluated for diagnostic accuracy at the individual patient level (Zikou et al., 2014; Thomann et al., 2017; Christodoulou et al., 2025). Compared to patients with irritable bowel syndrome (IBS), individuals with IBD exhibit more pronounced white matter microstructural damage and neuroinflammation-related abnormalities in some studies, suggesting that these imaging differences warrant further investigation as candidate biomarkers for differential diagnosis (Hong et al., 2014; Öhlmann et al., 2023). In the context of disease assessment, neuroimaging metrics show associations with clinical disease activity. Specifically, ACC functional connectivity strength and insular activation levels correlate with the Crohn’s Disease Activity Index (CDAI) and Mayo score. Additionally, the DTI-ALPS index in patients with UC and prefrontal gray matter volume alterations in patients with CD have been proposed as complementary indicators for assessing disease activity (Zhang et al., 2022; Thapaliya et al., 2023b; Thomann et al., 2024). Regarding prognostic prediction, baseline neuroimaging abnormalities have shown associations with symptom recurrence and disease severity in several studies. Patients exhibiting baseline ACC functional abnormalities or reduced hippocampal gray matter volume have been linked to higher recurrence risk. In patients with CD, baseline DMN disruption has been associated with long-term prognosis, while in patients with UC, baseline hippocampal abnormalities have been associated with long-term emotional disturbance and disease recurrence (Thomann et al., 2017; Fan et al., 2019; Nair et al., 2019; Yang et al., 2024a). In terms of treatment monitoring, small-scale studies have reported that anti-tumor necrosis factor (anti-TNF) therapy is associated with changes in pain matrix activity and neuroinflammatory markers, which correlate with symptomatic relief (Hess et al., 2015; Sergeeva et al., 2015; Gray et al., 2018). Similarly, preliminary neuromodulatory studies have reported that changes in network-level connectivity correlate with improvements in emotional symptoms and abdominal pain (Bonaz et al., 2013; Neeb et al., 2019; D'haens et al., 2023). Collectively, these early findings provide proof-of-concept for the potential clinical utility of neuroimaging in IBD; however, none of the markers described have been independently validated for routine clinical use.

Despite these advances in identifying potential clinical applications, several significant barriers currently impede the translation of neuroimaging findings into routine IBD management. Qualitative appraisal of methodological quality across included studies revealed widespread limitations that weaken the robustness and generalizability of current evidence. Synthesis of the existing literature identifies several overarching research limitations: (1) Limited sample sizes and statistical power: The median sample size across the 175 included studies was approximately 25–35 patients per group, with fewer than 10 studies recruiting over 100 patients per group. Only a minority employed multi-center recruitment, limiting the generalizability and statistical power to detect subtle brain alterations. (2) Inadequate subtype differentiation: Systematic comparisons between CD and UC are scarce, and standardization for confounders like disease activity and duration is lacking, compromising cross-study comparability. (3) Predominance of cross-sectional designs: The vast majority of studies were cross-sectional, with only a small minority employing longitudinal designs, precluding inferences about the temporal dynamics of brain changes and causal brain-gut relationships. (4) Lack of analytical standardization: Variability in acquisition, preprocessing, and analytical protocols across studies hinders data integration and meta-analysis. (5) Absence of validated imaging biomarkers: Neuroimaging markers remain insufficiently validated in large cohorts, and standardized assessment frameworks are lacking, impeding routine clinical translation. Collectively, these methodological constraints indicate that the current evidence base should be interpreted as indicative rather than definitive; direct replication studies remain exceedingly rare, and the field is not yet at a stage where individual neuroimaging markers can inform clinical decision-making.

## 4 Discussion

This study represents the first integrated use of bibliometric analysis and literature review to delineate the evolution, core themes, and knowledge architecture of neuroimaging research in IBD over the past two decades. By combining macroscopic mapping with microscopic evidence synthesis, we not only document the rapid growth of this field but also reveal its intrinsic structure and theoretical logic, providing multi-level support for understanding IBD as a prototypical disorder of brain-gut interaction.

### 4.1 Synthesis of core findings and emerging research frontiers

First, from a macroscopic bibliometric perspective, IBD neuroimaging research has evolved from early exploratory efforts into a phase of rapid expansion and increasing theoretical convergence around the brain-gut axis as an organizing framework. The annual publication trend shows accelerated growth since 2021, with 27 articles published by 2025, reflecting heightened academic interest in brain-gut interactions. Keyword burst analysis further captures this evolution: early bursts centered on descriptive terms such as “inflammation” and “Crohn’s disease,” whereas recent bursts feature integrative concepts like “brain-gut axis” (strength 3.31, ongoing through 2025) and “DMN” (strength 2.04, 2022–2023). Country collaboration networks identify the United States as a global hub (total link strength 32.00), with deep collaborations with France (average citations 163.78) and Canada (average citations 54.25) producing foundational work, such as Bonaz et al.(Bonaz and Bernstein, 2013) in *Gastroenterology* (586 citations), which anchored the brain-gut interaction framework. The dual-map overlay (Figure 5B) provides a macroscopic visualization of this interdisciplinary knowledge transfer, revealing prominent citation trajectories from molecular biology and immunology to clinical gastroenterology and neuroscience—a pattern that mirrors the biological reality that IBD neuroimaging research is fundamentally built upon the translation of basic immune and microbiological discoveries into brain-gut interaction studies. Together, these bibliometric findings suggest that the field is progressively organizing around the brain-gut axis as a core theoretical construct, moving beyond its earlier phase of fragmented observations. At the same time, the narrative synthesis reveals that the empirical testing of brain-gut pathways—particularly studies simultaneously assessing neural, immune, and microbiota measures—lags considerably behind the theoretical centrality of the brain-gut axis in the literature.

Second, by aligning bibliometric hotspot identification with in-depth literature synthesis, this study provides converging evidence that brain alterations in IBD exhibit a pronounced “network-based” pattern and distinct subtype-specific signatures. Keyword co-occurrence analysis identified two major clusters—"brain regions/networks” (e.g., ACC, insula, DMN) and “clinical symptoms” (e.g., abdominal pain, anxiety, depression)—visually depicted in Figure 7A, underscoring a central research question: how regional brain abnormalities relate to core clinical manifestations. The literature review further reveals that the available evidence indicates that these abnormalities predominantly converge within an emotional and interoceptive network comprising the ACC, insula, and amygdala (Bao et al., 2016; Fan et al., 2020; Kong et al., 2024). Structural (GMV reduction) and functional (FC alterations) changes in this network show consistent correlations with abdominal pain intensity and anxiety/depression scores (Bao et al., 2015; Kong et al., 2021; Zhang et al., 2025). Notably, CD and UC emerge as distinct keywords, reflecting subtype-focused inquiry. The literature review deepens this distinction: CD is characterized by hyperactivity in anterior cognitive-affective networks (consistent with frequent keywords such as “ACC” and “prefrontal cortex”)(Hou et al., 2020; Kong et al., 2021; Kornelsen et al., 2021), whereas UC predominantly involves posterior memory-related regions (hippocampus) and sensory network disruptions (linked to “hippocampus,” “visual network”)(Fan et al., 2019; Öhlmann et al., 2023). This convergent–divergent pattern raises the hypothesis that CD and UC may engage different neuroimmune pathways to affect the brain, ultimately converging on shared symptomatic networks, though direct evidence testing this proposition remains limited (Christodoulou et al., 2025). Moreover, certain brain changes persist during remission, suggesting they represent trait-like disease markers rather than mere state-dependent phenomena (Nair et al., 2019; Hou et al., 2020). This macro–micro correspondence—where bibliometric clusters are substantiated by detailed narrative evidence in Table 8, yet the narrative synthesis also reveals nuances such as variability in effect direction that bibliometric maps cannot capture—underscores the complementary value of the dual approach and offers a working framework for understanding the neural basis of IBD heterogeneity, though its generality awaits testing in larger, more representative samples (Christodoulou et al., 2025; Yin et al., 2026).

Third, through analysis of highly cited references and core author networks, this study maps the field’s intellectual foundations and emerging pathways toward clinical translation. Reference co-citation analysis identifies Agostini et al. (2013) and Bao et al. (2015) as methodological and theoretical cornerstones—the former establishing VBM paradigms in IBD brain structure research, the latter revealing emotion-related brain abnormalities. Author collaboration networks highlight two research paradigms: Liu Peng’s team (13 publications) exemplifies intensive domestic collaboration focused on multimodal imaging technique development (Chen et al., 2023; Li P. et al., 2024), while Bernstein’s team (average citations 72.7) reflects sustained international collaboration advancing brain-gut axis research translation (Kornelsen et al., 2020; Kornelsen et al., 2021, 2022). Clinical trial analysis and literature review delineate key translational directions: (1) Auxiliary diagnosis and differential diagnosis (Zikou et al., 2014; Thomann et al., 2017; Christodoulou et al., 2025); (2) Disease activity and prognosis assessment (Thomann et al., 2017; Fan et al., 2019; Nair et al., 2019; Yang et al., 2024a); (3) Treatment response monitoring (Hess et al., 2015; Sergeeva et al., 2015; Gray et al., 2018). However, systematic limitations—small sample sizes, methodological heterogeneity, predominance of cross-sectional designs, and lack of standardized imaging biomarkers—remain major barriers to clinical translation, resulting in fragmented and poorly reproducible findings. This macro–micro correspondence reveals a telling pattern: while bibliometric data show a field rapidly coalescing around the brain-gut axis framework, the narrative synthesis demonstrates that studies directly testing this framework remain disproportionately scarce relative to descriptive mapping studies—a gap already noted above that represents both a challenge and a clear direction for the next phase of research.

Fourth, keyword timeline analysis and frontier detection jointly point toward future research directions. Thematic evolution, visualized in the keyword timeline (Figure 7C), shows a clear trajectory: from disease-localization mapping (pre-2010), to resting-state networks and psychiatric comorbidities (2015–2020), and more recently to multimodal integration and brain-gut mechanisms (2020–present). This temporal progression mirrors the biological maturation of the field: early studies asked whether the IBD brain differs structurally; mid-period studies asked how these differences relate to specific symptoms and networks; current studies increasingly ask why these differences arise, invoking brain-gut pathways and neuroinflammation as explanatory frameworks. Burst analysis identifies emerging frontiers, including “multimodal” (2022–2025), “functional connectivity” (2023–2025), and “neuroinflammation” (2024–2025), suggesting several priority directions: (1) large-scale, multi-center studies with standardized imaging protocols to enhance reproducibility and generalizability; (2) longitudinal and interventional designs (e.g., neuromodulation, anti-inflammatory therapy) to establish causal brain-gut dynamics; (3) deep multimodal data fusion integrating structural, functional, and molecular imaging with omics and immune markers to construct individualized brain-gut models; and (4) subtype- and symptom-specific analyses leveraging advanced network methods and machine learning to explore whether imaging “fingerprints” predictive of clinical outcomes and treatment response can be identified, with rigorous external validation as a prerequisite for any clinical consideration.

### 4.2 Implications for future research and clinical practice

The integrated findings of this study have several important implications for both research design and clinical translation. For future research, there is an urgent need to move beyond small-scale, cross-sectional observations toward multi-center, longitudinal studies with deep phenotyping. Incorporating neuroimaging metrics as secondary or exploratory endpoints in clinical trials would enable systematic evaluation of their utility in capturing treatment effects on brain–gut dynamics. For clinical practice, the distinct neuroimaging profiles identified for CD and UC, as well as their associations with specific symptom clusters, suggest the potential for developing subtype- and symptom-specific imaging assessment strategies. For instance, in patients with CD in remission who present with severe fatigue as the predominant symptom, targeted evaluation of DMN function may provide valuable insights into the central contributions to this debilitating condition, potentially guiding personalized intervention approaches such as neuromodulation.

### 4.3 Limitations

Several limitations should be acknowledged. First, the literature search was restricted to Web of Science Core Collection and Scopus, which, despite covering major English-language journals, may omit conference proceedings, books, or non-English publications. Second, screening based on titles and abstracts may introduce selection bias. Third, bibliometric methods cannot assess study quality, and keyword standardization may involve semantic merging error. Fourth, due to the descriptive and exploratory nature of this review, a formal risk of bias assessment (e.g., using Cochrane tools) was not performed. To compensate, we employed bibliometric indicators as proxies for scientific influence and conducted a systematic qualitative appraisal of key methodological limitations across the included studies (e.g., sample size, study design, analytical heterogeneity), as detailed in Section 3.2.5. Finally, the search cutoff in early 2026 may not capture the most recent publications. Future studies could expand data sources, incorporate full-text review, and introduce quality assessment metrics to deepen and broaden the analysis.

## 5 Conclusion

This study provides a comprehensive integrated bibliometric and systematic review of neuroimaging research in IBD, mapping the field’s evolution, core themes, and knowledge structure over two decades. Bibliometric findings reveal rapid growth since 2021, with China and the United States as leading contributors, and the “brain–gut axis” and “DMN” as central frontiers. Literature synthesis provides consistent evidence that brain alterations predominantly converge on an emotional–interoceptive network (ACC–insula–amygdala), showing close associations with abdominal pain, anxiety, and depression, and with distinguishable neuroimaging phenotypes for CD and UC emerging from the available data. Although neuroimaging findings show exploratory associations with clinical parameters relevant to diagnosis, activity assessment, and treatment monitoring, significant bottlenecks remain—small sample sizes, methodological heterogeneity, and a scarcity of longitudinal data—that currently preclude direct clinical application. Future efforts should prioritize multi-center validation, longitudinal designs to test mechanistic hypotheses, and multimodal integration to advance the field from descriptive observation toward a rigorous evidence base suitable for eventual clinical translation.

## Data Availability

The original contributions presented in the study are included in the article/Supplementary material, further inquiries can be directed to the corresponding authors.
