# Supplementary material for: Mapping the neuroimaging landscape of inflammatory bowel disease: a bibliometric analysis and systematic scoping review
Source: Front Neurosci. 2026 Jun 4;20:1821831. doi: 10.3389/fnins.2026.1821831 (PMC13275236; doi:10.3389/fnins.2026.1821831)
Supplement: Supplementary file 1 [file Table_1.DOC]

1. **Web of Science Core Database search strategy：**

TS=("inflammatory bowel disease*" OR "IBD" OR "ulcerative colitis" OR "Crohn* disease") AND TS=(("neuroimag*" OR "neurofunctional imag*" OR "functional neuroimag*" OR "functional brain imag*") OR ("magnetic resonance imag*" OR MRI OR "functional magnetic resonance imag*" OR "fMRI" OR "resting state" OR "functional connectivity" OR "BOLD" OR "Blood Oxygen Level Dependent" OR "MRS" OR "magnetic resonance spectroscopy" OR "DTI" OR "diffusion tensor imag*" OR "ASL" OR "arterial spin labeling" OR "DWI" OR "Diffusion Weighted Imag*" OR "QSM" OR "Quantitative Susceptibility Mapping" OR "SWI" OR "Susceptibility Weighted Imag*") OR ("functional near infrared spectroscopy" OR "fNIRS" OR "cortical activit*") OR ("EEG" OR "electroencephalograph*") OR ("MEG" OR "magnetoencephalograph*") OR ("PET" OR "positron emission tomograph*" OR "SPECT" OR "single photon emission computed tomograph*")) AND TS=("brain" OR "cerebral" OR "neuro*" OR "cognit*" OR "mental" OR "central nervous system" OR "CNS" OR "brain-gut" OR "gut-brain" OR "brain-gut axis" OR "gut-brain axis" OR "neuroimmune")

1. **Scopus Database search strategy：**

(TITLE-ABS-KEY("inflammatory bowel disease*" OR "IBD" OR "ulcerative colitis" OR "Crohn* disease") AND TITLE-ABS-KEY(("neuroimag*" OR "neurofunctional imag*" OR "functional neuroimag*" OR "functional brain imag*") OR ("magnetic resonance imag*" OR MRI OR "functional magnetic resonance imag*" OR "fMRI" OR "resting state" OR "functional connectivity" OR "BOLD" OR "Blood Oxygen Level Dependent" OR "MRS" OR "magnetic resonance spectroscopy" OR "DTI" OR "diffusion tensor imag*" OR "ASL" OR "arterial spin labeling" OR "DWI" OR "Diffusion Weighted Imag*" OR "QSM" OR "Quantitative Susceptibility Mapping" OR "SWI" OR "Susceptibility Weighted Imag*") OR ("functional near infrared spectroscopy" OR "fNIRS" OR "cortical activit*") OR ("EEG" OR "electroencephalograph*") OR ("MEG" OR "magnetoencephalograph*") OR ("PET" OR "positron emission tomograph*" OR "SPECT" OR "single photon emission computed tomograph*")) AND TITLE-ABS-KEY("brain" OR "cerebral" OR "neuro*" OR "cognit*" OR "mental" OR "central nervous system" OR "CNS" OR "brain-gut" OR "gut-brain" OR "brain-gut axis" OR "gut-brain axis" OR "neuroimmune")) AND (PUBYEAR AFT 1999)

1. **Pubmed clinical trials search strategy：**

Search: (((("inflammatory bowel disease*"[Title/Abstract] OR IBD[Title/Abstract] OR "ulcerative colitis"[Title/Abstract] OR "Crohn* disease"[Title/Abstract]))) AND ((("neuroimag*"[Title/Abstract] OR "neurofunctional imag*"[Title/Abstract] OR "functional neuroimag*"[Title/Abstract] OR "functional brain imag*"[Title/Abstract]) OR ("magnetic resonance imag*"[Title/Abstract] OR MRI[Title/Abstract] OR "functional magnetic resonance imag*"[Title/Abstract] OR fMRI[Title/Abstract] OR "resting state"[Title/Abstract] OR "functional connectivity"[Title/Abstract] OR BOLD[Title/Abstract] OR "Blood Oxygen Level Dependent"[Title/Abstract] OR "MRS"[Title/Abstract] OR "magnetic resonance spectroscopy"[Title/Abstract] OR "DTI"[Title/Abstract] OR "diffusion tensor imag*"[Title/Abstract] OR ASL[Title/Abstract] OR "arterial spin labeling"[Title/Abstract] OR DWI[Title/Abstract] OR "Diffusion Weighted Imag*"[Title/Abstract] OR QSM[Title/Abstract] OR "Quantitative Susceptibility Mapping"[Title/Abstract] OR SWI[Title/Abstract] OR "Susceptibility Weighted Imag*"[Title/Abstract]) OR ("functional near infrared spectroscopy"[Title/Abstract] OR fNIRS[Title/Abstract] OR "cortical activit*"[Title/Abstract]) OR (EEG[Title/Abstract] OR electroencephalograph*[Title/Abstract]) OR (MEG[Title/Abstract] OR magnetoencephalograph*[Title/Abstract]) OR (PET[Title/Abstract] OR "positron emission tomograph*"[Title/Abstract] OR "SPECT"[Title/Abstract] OR "single photon emission computed tomograph*"[Title/Abstract])))) AND ((("brain"[Title/Abstract] OR "cerebral"[Title/Abstract] OR "neuro*"[Title/Abstract] OR "cognit*"[Title/Abstract] OR "mental"[Title/Abstract] OR "central nervous system"[Title/Abstract] OR CNS[Title/Abstract] OR "brain-gut"[Title/Abstract] OR "gut-brain"[Title/Abstract] OR "brain-gut axis"[Title/Abstract] OR "gut-brain axis"[Title/Abstract] OR "neuroimmune"[Title/Abstract]))) Filters: Clinical Trial, from 2000/1/1 - 2026/1/27 Sort by: Publication Date

**Pubmed**

A total of 7 clinical articles were identified through PubMed screening.

PMID- 30905546

OWN - NLM

STAT- MEDLINE

DCOM- 20191206

LR - 20191217

IS - 1876-4754 (Electronic)

IS - 1876-4754 (Linking)

VI - 12

IP - 4

DP - 2019 Jul-Aug

TI - Transcranial direct current stimulation in inflammatory bowel disease patients

modifies resting-state functional connectivity: A RCT.

PG - 978-980

LID - S1935-861X(19)30080-4 [pii]

LID - 10.1016/j.brs.2019.03.001 [doi]

AB - BACKGROUND: Chronic pain is known to be associated with functional and structural

changes in the brain. Inflammatory bowel disease (IBD) presents with chronic

abdominal pain in almost 35% of all patients. This study investigates structural

and functional changes in magnetic resonance imaging (MRI) after transcranial

direct current stimulation (tDCS) applied to ameliorate pain in IBD. METHODS:

This phase-III, placebo-controlled, randomized study included 36 patients with

IBD and chronic pain. MRI scans were performed before and following tDCS, which

was applied for 5 days. RESULTS/CONCLUSION: For the first time, this study

revealed an association of changes in resting-state functional MRI and pain

reduction in IBD. There was a significant increase in functional connectivity

after active tDCS within the visual medial and the right frontoparietal network

being connected with the amygdala, the insula, and the primary somatosensory

cortex indicating central pain mechanisms in IBD. Moreover, tDCS offers a novel

therapeutic strategy for abdominal pain.

CI - Copyright © 2019 The Authors. Published by Elsevier Inc. All rights reserved.

FAU - Neeb, Lars

AU - Neeb L

AD - Department of Neurology, Charité - Universitätsmedizin Berlin, Corporate Member

of Freie Universität Berlin, Humboldt-Universität zu Berlin, and Berlin Institute

of Health, Berlin, Germany. Electronic address: Lars.neeb@charite.de.

FAU - Bayer, Arian

AU - Bayer A

AD - Medizinische Klinik M. S. Gastroenterologie, Infektiologie und Rheumatologie,

Charité -Universitätsmedizin Berlin, Corporate Member of Freie Universität

Berlin, Humboldt-Universität zu Berlin, and Berlin Institute of Health, Berlin,

Germany. Electronic address: arian.bayer@charite.de.

FAU - Bayer, Kian-Elias

AU - Bayer KE

AD - Medizinische Klinik M. S. Gastroenterologie, Infektiologie und Rheumatologie,

Charité -Universitätsmedizin Berlin, Corporate Member of Freie Universität

Berlin, Humboldt-Universität zu Berlin, and Berlin Institute of Health, Berlin,

Germany. Electronic address: kian-elias.bayer@charite.de.

FAU - Farmer, Annabelle

AU - Farmer A

AD - Medizinische Klinik M. S. Gastroenterologie, Infektiologie und Rheumatologie,

Charité -Universitätsmedizin Berlin, Corporate Member of Freie Universität

Berlin, Humboldt-Universität zu Berlin, and Berlin Institute of Health, Berlin,

Germany. Electronic address: annabelle.farmer@charite.de.

FAU - Fiebach, Jochen B

AU - Fiebach JB

AD - Center for Stroke Research Berlin, Charité - Universitätsmedizin Berlin,

Corporate Member of Freie Universität Berlin, Humboldt-Universität zu Berlin, and

Berlin Institute of Health, Germany. Electronic address:

jochen.fiebach@charite.de.

FAU - Siegmund, Britta

AU - Siegmund B

AD - Medizinische Klinik M. S. Gastroenterologie, Infektiologie und Rheumatologie,

Charité -Universitätsmedizin Berlin, Corporate Member of Freie Universität

Berlin, Humboldt-Universität zu Berlin, and Berlin Institute of Health, Berlin,

Germany. Electronic address: britta.siegmund@charite.de.

FAU - Volz, Magdalena Sarah

AU - Volz MS

AD - Medizinische Klinik M. S. Gastroenterologie, Infektiologie und Rheumatologie,

Charité -Universitätsmedizin Berlin, Corporate Member of Freie Universität

Berlin, Humboldt-Universität zu Berlin, and Berlin Institute of Health, Berlin,

Germany; Berlin Institute of Health, 10178, Berlin, Germany. Electronic address:

magdalena.pruess@charite.de.

LA - eng

PT - Clinical Trial, Phase III

PT - Journal Article

PT - Randomized Controlled Trial

PT - Research Support, Non-U.S. Gov't

DEP - 20190305

PL - United States

TA - Brain Stimul

JT - Brain stimulation

JID - 101465726

SB - IM

MH - Adult

MH - Brain/*diagnostic imaging/physiology

MH - Double-Blind Method

MH - Female

MH - Humans

MH - Inflammatory Bowel Diseases/*diagnostic imaging/*therapy

MH - Magnetic Resonance Imaging/*methods

MH - Male

MH - Middle Aged

MH - Nerve Net/*diagnostic imaging/physiology

MH - Rest/physiology

MH - Transcranial Direct Current Stimulation/*methods

OTO - NOTNLM

OT - Inflammatory bowel disease

OT - Magnetic resonance imaging

OT - Transcranial direct current stimulation

OT - Visceral pain

EDAT- 2019/03/25 06:00

MHDA- 2019/12/18 06:00

CRDT- 2019/03/26 06:00

PHST- 2019/01/03 00:00 [received]

PHST- 2019/02/26 00:00 [revised]

PHST- 2019/03/01 00:00 [accepted]

PHST- 2019/03/25 06:00 [pubmed]

PHST- 2019/12/18 06:00 [medline]

PHST- 2019/03/26 06:00 [entrez]

AID - S1935-861X(19)30080-4 [pii]

AID - 10.1016/j.brs.2019.03.001 [doi]

PST - ppublish

SO - Brain Stimul. 2019 Jul-Aug;12(4):978-980. doi: 10.1016/j.brs.2019.03.001. Epub

2019 Mar 5.

PMID- 23582152

OWN - NLM

STAT- MEDLINE

DCOM- 20140124

LR - 20211021

IS - 1872-6623 (Electronic)

IS - 0304-3959 (Print)

IS - 0304-3959 (Linking)

VI - 154

IP - 7

DP - 2013 Jul

TI - Diminished neurokinin-1 receptor availability in patients with two forms of

chronic visceral pain.

PG - 987-96

LID - S0304-3959(13)00082-1 [pii]

LID - 10.1016/j.pain.2013.02.026 [doi]

AB - Central sensitization and dysregulation of peripheral substance P and

neurokinin-1 receptor (NK-1R) signaling are associated with chronic abdominal

pain in inflammatory bowel disease (IBD) and irritable bowel syndrome (IBS).

Although positron emission tomography (PET) has demonstrated that patients with

injury-related chronic pain have diminished NK-1R availability in the brain, it

is unknown whether these deficits are present in IBD and IBS patients, who have

etiologically distinct forms of non-injury-related chronic pain. This study's aim

was to determine if patients with IBD or IBS exhibit deficits in brain expression

of NK-1Rs relative to healthy controls (HCs), the extent to which expression

patterns differ across patient populations, and if these patterns differentially

relate to clinical parameters. PET with [(18)F]SPA-RQ was used to measure NK-1R

availability by quantifying binding potential (BP) in the 3 groups. Exploratory

correlation analyses were performed to detect associations between NK-1R BP and

physical symptoms. Compared to HCs, IBD patients had NK-1R BP deficits across a

widespread network of cortical and subcortical regions. IBS patients had similar,

but less pronounced deficits. BP in a subset of these regions was robustly

related to discrete clinical parameters in each patient population. Widespread

deficits in NK-1R BP occur in IBD and, to a lesser extent, IBS; however, discrete

clinical parameters relate to NK-1R BP in each patient population. This suggests

that potential pharmacological interventions that target NK-1R signaling may be

most effective for treating distinct symptoms in IBD and IBS.

CI - Copyright © 2013 International Association for the Study of Pain. Published by

Elsevier B.V. All rights reserved.

FAU - Jarcho, Johanna M

AU - Jarcho JM

AD - Section on Developmental and Affective Neuroscience, National Institute of Mental

Health, Bethesda, MD, USA.

FAU - Feier, Natasha A

AU - Feier NA

FAU - Bert, Alberto

AU - Bert A

FAU - Labus, Jennifer A

AU - Labus JA

FAU - Lee, Maunoo

AU - Lee M

FAU - Stains, Jean

AU - Stains J

FAU - Ebrat, Bahar

AU - Ebrat B

FAU - Groman, Stephanie M

AU - Groman SM

FAU - Tillisch, Kirsten

AU - Tillisch K

FAU - Brody, Arthur L

AU - Brody AL

FAU - London, Edythe D

AU - London ED

FAU - Mandelkern, Mark A

AU - Mandelkern MA

FAU - Mayer, Emeran A

AU - Mayer EA

LA - eng

GR - T32 MH017140/MH/NIMH NIH HHS/United States

GR - F31 DA021951/DA/NIDA NIH HHS/United States

GR - F31 DA028812/DA/NIDA NIH HHS/United States

GR - R24 AT002681/AT/NCCIH NIH HHS/United States

GR - T32-MH017140/MH/NIMH NIH HHS/United States

GR - F31-DA021951/DA/NIDA NIH HHS/United States

GR - AT00268/AT/NCCIH NIH HHS/United States

GR - R01 DA020872/DA/NIDA NIH HHS/United States

GR - I01 CX000412/CX/CSRD VA/United States

GR - R01 DA20872/DA/NIDA NIH HHS/United States

GR - F313 DA028812/DA/NIDA NIH HHS/United States

PT - Comparative Study

PT - Controlled Clinical Trial

PT - Journal Article

PT - Research Support, N.I.H., Extramural

PT - Research Support, Non-U.S. Gov't

PT - Research Support, U.S. Gov't, Non-P.H.S.

DEP - 20130305

PL - United States

TA - Pain

JT - Pain

JID - 7508686

RN - 0 (Receptors, Neurokinin-1)

SB - IM

CIN - Pain. 2013 Jul;154(7):966-967. doi: 10.1016/j.pain.2013.04.020. PMID: 23643331

MH - Adult

MH - Brain/*metabolism

MH - Chronic Pain

MH - Down-Regulation

MH - Female

MH - Humans

MH - Inflammatory Bowel Diseases/*metabolism

MH - Irritable Bowel Syndrome/*metabolism

MH - Male

MH - Pilot Projects

MH - Receptors, Neurokinin-1/*metabolism

MH - Tissue Distribution

MH - Visceral Pain/*metabolism

PMC - PMC4294187

MID - NIHMS603671

COIS- Conflict of interest statement: The authors report no conflict of interest.

EDAT- 2013/04/16 06:00

MHDA- 2014/01/25 06:00

PMCR- 2015/01/14

CRDT- 2013/04/16 06:00

PHST- 2012/12/12 00:00 [received]

PHST- 2013/02/08 00:00 [revised]

PHST- 2013/02/22 00:00 [accepted]

PHST- 2013/04/16 06:00 [entrez]

PHST- 2013/04/16 06:00 [pubmed]

PHST- 2014/01/25 06:00 [medline]

PHST- 2015/01/14 00:00 [pmc-release]

AID - S0304-3959(13)00082-1 [pii]

AID - 10.1016/j.pain.2013.02.026 [doi]

PST - ppublish

SO - Pain. 2013 Jul;154(7):987-96. doi: 10.1016/j.pain.2013.02.026. Epub 2013 Mar 5.

PMID- 40658641

OWN - NLM

STAT- MEDLINE

DCOM- 20251121

LR - 20251216

IS - 1536-4844 (Electronic)

IS - 1078-0998 (Print)

IS - 1078-0998 (Linking)

VI - 31

IP - 11

DP - 2025 Nov 1

TI - Group-Delivered Mindfulness-Based Cognitive Therapy to Reduce Psychological

Distress and Improve Sleep in Patients With Inflammatory Bowel Diseases: A

Multicenter Randomized Controlled Trial (MindIBD).

PG - 3021-3032

LID - 10.1093/ibd/izaf116 [doi]

AB - BACKGROUND: Many patients with inflammatory bowel disease (IBD) suffer from

psychological distress, sleep disturbances, fatigue, and a reduced quality of

life-even during remission. Mindfulness-Based Cognitive Therapy (MBCT) has been

effective in other populations, and therefore it may also benefit IBD patients.

The primary objective was to evaluate the effectiveness of MBCT plus treatment as

usual (TAU) in reducing psychological distress compared to TAU alone. METHODS:

This multicenter randomized controlled trial study included IBD patients in

remission, aged 16 and older, who experienced at least mild levels of

psychological distress (Hospital Anxiety and Depression Scale ≥ 11). Assessments

were conducted at baseline, post-intervention (3 months), and at 6, 9, and 12

months after baseline. This trial was registered at ClinicalTrials.gov:

NCT04646785. RESULTS: A total of 142 participants were allocated to MBCT + TAU

(n = 70) or TAU alone (n = 72). Group-delivered MBCT significantly reduced

psychological distress (d = -0.61) and improved well-being (d = 0.40)

post-intervention, with consolidation of the effects over time. Exploratory

objective sleep metrics (ie, electroencephalography) showed a reduction in total

sleep time (d = 0.67) after MBCT with an increase in the proportion of deep sleep

(d = 0.70). While flare occurrence showed no difference, fecal calprotectin

levels reduced in the MBCT + TAU group over the follow-up period (d = -0.49).

CONCLUSIONS: Mindfulness-Based Cognitive Therapy can be considered a valuable

addition to the limited effective psychosocial interventions set for IBD

patients, as it reduces psychological distress and improves well-being. This

study also shows the possible impact of MBCT on biological processes, such as

sleep and inflammation.

CI - © 2025 Crohn’s & Colitis Foundation. Published by Oxford University Press on

behalf of Crohn’s & Colitis Foundation.

FAU - Ter Avest, Milou M

AU - Ter Avest MM

AUID- ORCID: 0000-0002-8817-7817

AD - Department of Psychiatry, Centre for Mindfulness, Radboud University Medical

Centre, Nijmegen, the Netherlands.

AD - Donders Institute for Brain, Cognition and Behaviour, Radboud University Medical

Centre, Nijmegen, the Netherlands.

AD - Department of Gastroenterology and Hepatology, Jeroen Bosch Hospital,

's-Hertogenbosch, the Netherlands.

FAU - Huijbers, Marloes J

AU - Huijbers MJ

AD - Department of Psychiatry, Centre for Mindfulness, Radboud University Medical

Centre, Nijmegen, the Netherlands.

FAU - Horjus, Carmen S

AU - Horjus CS

AD - Department of Gastroenterology and Hepatology, Rijnstate Hospital, Arnhem, the

Netherlands.

FAU - Römkens, Tessa E H

AU - Römkens TEH

AD - Department of Gastroenterology and Hepatology, Jeroen Bosch Hospital,

's-Hertogenbosch, the Netherlands.

FAU - Witteman, Ellen M

AU - Witteman EM

AD - Department of Gastroenterology and Hepatology, Canisius Wilhelmina Hospital,

Nijmegen, the Netherlands.

FAU - van Dop, Willemijn A

AU - van Dop WA

AD - Department of Gastroenterology and Hepatology, Radboud University Medical Centre,

Nijmegen, the Netherlands.

FAU - Dresler, Martin

AU - Dresler M

AUID- ORCID: 0000-0001-7441-3818

AD - Donders Institute for Brain, Cognition and Behaviour, Radboud University Medical

Centre, Nijmegen, the Netherlands.

FAU - Donders, A Rogier T

AU - Donders ART

AD - Department IQ Health, Radboud University Medical Centre, Radboud Institute for

Health Sciences, Nijmegen, the Netherlands.

FAU - Dijkstra, Gerard

AU - Dijkstra G

AD - Department of Gastroenterology and Hepatology, University Medical Centre

Groningen, University of Groningen, Groningen, the Netherlands.

FAU - Nissen, Loes H C

AU - Nissen LHC

AD - Department of Gastroenterology and Hepatology, Jeroen Bosch Hospital,

's-Hertogenbosch, the Netherlands.

FAU - Speckens, Anne E M

AU - Speckens AEM

AD - Department of Psychiatry, Centre for Mindfulness, Radboud University Medical

Centre, Nijmegen, the Netherlands.

AD - Donders Institute for Brain, Cognition and Behaviour, Radboud University Medical

Centre, Nijmegen, the Netherlands.

LA - eng

SI - ClinicalTrials.gov/NCT04646785

GR - 555003201/Netherlands Organization for Health Research and Development/

GR - MindMore Foundation/

GR - ZONMW_/ZonMw/Netherlands

PT - Journal Article

PT - Multicenter Study

PT - Randomized Controlled Trial

PL - England

TA - Inflamm Bowel Dis

JT - Inflammatory bowel diseases

JID - 9508162

SB - IM

MH - Humans

MH - Female

MH - *Mindfulness/methods

MH - Male

MH - Adult

MH - *Cognitive Behavioral Therapy/methods

MH - *Inflammatory Bowel Diseases/psychology/complications/therapy

MH - *Psychological Distress

MH - Quality of Life

MH - Middle Aged

MH - *Sleep Wake Disorders/therapy/etiology

MH - Young Adult

MH - *Stress, Psychological/therapy/etiology

MH - *Psychotherapy, Group/methods

MH - Depression/therapy/etiology

MH - Anxiety/therapy

MH - Treatment Outcome

MH - Adolescent

MH - Follow-Up Studies

PMC - PMC12638053

OAB - Inflammatory bowel diseases are associated with a high psychological burden with

currently limited psychological treatment options. This randomized controlled

trial shows that mindfulness-based cognitive therapy can be added as an effective

treatment option to reduce psychological distress.

OABL- eng

OTO - NOTNLM

OT - Mindfulness-Based Cognitive Therapy

OT - inflammatory bowel diseases

OT - psychological distress

OT - randomized controlled trial

COIS- L.N. has served on the advisory boards of Ferring, AbbVie, and Johnson & Johnson,

and G.D. received a grant from Royal DSM and served on the advisory board of

Pharmacosmos; both disclosures are unrelated to the published work. All the other

authors declare that they have no conflicts of interest.

EDAT- 2025/07/14 18:27

MHDA- 2025/11/21 18:25

PMCR- 2025/07/14

CRDT- 2025/07/14 13:23

PHST- 2025/01/16 00:00 [received]

PHST- 2025/11/21 18:25 [medline]

PHST- 2025/07/14 18:27 [pubmed]

PHST- 2025/07/14 13:23 [entrez]

PHST- 2025/07/14 00:00 [pmc-release]

AID - 8200868 [pii]

AID - izaf116 [pii]

AID - 10.1093/ibd/izaf116 [doi]

PST - ppublish

SO - Inflamm Bowel Dis. 2025 Nov 1;31(11):3021-3032. doi: 10.1093/ibd/izaf116.

PMID- 27857211

OWN - NLM

STAT- MEDLINE

DCOM- 20180516

LR - 20230727

IS - 2045-2322 (Electronic)

IS - 2045-2322 (Linking)

VI - 6

DP - 2016 Nov 18

TI - Different brain responses to electro-acupuncture and moxibustion treatment in

patients with Crohn's disease.

PG - 36636

LID - 10.1038/srep36636 [doi]

LID - 36636

AB - This study aimed to investigate changes in resting state brain activity in

remissive Crohn's Disease (CD) patients after electro-acupuncture or moxibustion

treatment. Fifty-two CD patients and 36 healthy subjects were enrolled, and 36

patients were equally and randomly assigned to receive either electro-acupuncture

or moxibustion treatment for twelve weeks. We used resting state functional

magnetic resonance imaging to assess Regional Homogeneity (ReHo) levels, and

Crohn's Disease Activity Index (CDAI) and Inflammatory Bowel Disease

Questionnaire (IBDQ) scores to evaluate disease severity and quality of life. The

results show that (i) The ReHo levels in CD patients were significantly increased

in cortical but decreased in subcortical areas, and the coupling between them was

declined. (ii) Both treatments decreased CDAI, increased IBDQ scores, and

normalized the ReHo values of the cortical and subcortical regions. (iii) ReHo

changes in multiple cortical regions were significantly correlated with CDAI

score decreases. ReHo changes in several subcortical regions in the

electro-acupuncture group, and those of several cortical regions in the

moxibustion group, were correlated with reduced CDAI. These findings suggest that

both treatments improved cortex-subcortical coupling in remissive CD patients,

but electro-acupuncture regulated homeostatic afferent processing network, while

moxibustion mainly regulated the default mode network of the brain.

FAU - Bao, Chunhui

AU - Bao C

AD - Key Laboratory of Acupuncture and Immunological Effects, Shanghai University of

Traditional Chinese Medicine, Shanghai, 200030, China.

FAU - Liu, Peng

AU - Liu P

AD - Life Sciences Research Center, School of Life Sciences and Technology, Xidian

University, Xi'an, Shaanxi, 710071, China.

FAU - Liu, Huirong

AU - Liu H

AD - Outpatient Department, Shanghai Research Institute of Acupuncture and Meridian,

Shanghai University of Traditional Chinese Medicine, Shanghai, 200030, China.

FAU - Jin, Xiaoming

AU - Jin X

AD - Stark Neurosciences Research Institute, Indiana University School of Medicine,

Indianapolis, Indiana, 46202, USA.

FAU - Calhoun, Vince D

AU - Calhoun VD

AD - The Mind Research Network, Albuquerque, NM, 87131, USA.

AD - Department of Electrical and Computer Engineering, University of New Mexico,

Albuquerque, NM, 87131, USA.

FAU - Wu, Luyi

AU - Wu L

AD - Key Laboratory of Acupuncture and Immunological Effects, Shanghai University of

Traditional Chinese Medicine, Shanghai, 200030, China.

FAU - Shi, Yin

AU - Shi Y

AD - Outpatient Department, Shanghai Research Institute of Acupuncture and Meridian,

Shanghai University of Traditional Chinese Medicine, Shanghai, 200030, China.

FAU - Zhang, Jianye

AU - Zhang J

AD - Department of Radiology, Shanghai Mental Health Center, Shanghai Jiaotong

University School of Medicine, Shanghai, 200030, China.

FAU - Zeng, Xiaoqing

AU - Zeng X

AD - Department of Gastroenterology, Zhongshan Hospital, Fudan University, Shanghai,

200032, China.

FAU - Ma, Lili

AU - Ma L

AD - Endoscopy Center, Zhongshan Hospital, Fudan University, Shanghai, 200032, China.

FAU - Qin, Wei

AU - Qin W

AD - Life Sciences Research Center, School of Life Sciences and Technology, Xidian

University, Xi'an, Shaanxi, 710071, China.

FAU - Zhang, Jingzhi

AU - Zhang J

AD - Key Laboratory of Acupuncture and Immunological Effects, Shanghai University of

Traditional Chinese Medicine, Shanghai, 200030, China.

FAU - Liu, Xiaoming

AU - Liu X

AD - Life Sciences Research Center, School of Life Sciences and Technology, Xidian

University, Xi'an, Shaanxi, 710071, China.

FAU - Tian, Jie

AU - Tian J

AD - Life Sciences Research Center, School of Life Sciences and Technology, Xidian

University, Xi'an, Shaanxi, 710071, China.

FAU - Wu, Huangan

AU - Wu H

AD - Key Laboratory of Acupuncture and Immunological Effects, Shanghai University of

Traditional Chinese Medicine, Shanghai, 200030, China.

LA - eng

GR - P20 GM103472/GM/NIGMS NIH HHS/United States

PT - Journal Article

PT - Randomized Controlled Trial

PT - Research Support, N.I.H., Extramural

PT - Research Support, Non-U.S. Gov't

DEP - 20161118

PL - England

TA - Sci Rep

JT - Scientific reports

JID - 101563288

SB - IM

MH - Adult

MH - Brain/diagnostic imaging/*physiopathology

MH - Case-Control Studies

MH - Crohn Disease/diagnostic imaging/physiopathology/*therapy

MH - *Electroacupuncture

MH - Female

MH - Humans

MH - Magnetic Resonance Imaging

MH - Male

MH - *Moxibustion

MH - Young Adult

PMC - PMC5114555

EDAT- 2016/11/20 06:00

MHDA- 2018/05/17 06:00

PMCR- 2016/11/18

CRDT- 2016/11/19 06:00

PHST- 2016/08/09 00:00 [received]

PHST- 2016/10/17 00:00 [accepted]

PHST- 2016/11/19 06:00 [entrez]

PHST- 2016/11/20 06:00 [pubmed]

PHST- 2018/05/17 06:00 [medline]

PHST- 2016/11/18 00:00 [pmc-release]

AID - srep36636 [pii]

AID - 10.1038/srep36636 [doi]

PST - epublish

SO - Sci Rep. 2016 Nov 18;6:36636. doi: 10.1038/srep36636.

PMID- 32579403

OWN - NLM

STAT- MEDLINE

DCOM- 20200805

LR - 20220415

IS - 1748-880X (Electronic)

IS - 0007-1285 (Print)

IS - 0007-1285 (Linking)

VI - 93

IP - 1112

DP - 2020 Aug

TI - Comparison of (18)F-FDG PET-MR and fecal biomarkers in the assessment of disease

activity in patients with ulcerative colitis.

PG - 20200167

LID - 10.1259/bjr.20200167 [doi]

LID - 20200167

AB - OBJECTIVE: To compare the diagnostic performance of fecal biomarkers and

(18)F-fludeoxyglucose ((18)F-FDG) positron emmision tomography-MR (PET-MR) in the

assessment of disease activity in patients with ulcerative colitis. METHODS: This

study was conducted under the framework of a single-center clinical trial

(clinicaltrials.gov [NCT03781284]). N = 50 participants were enrolled. Fecal

samples were collected before bowel preparation. All patients underwent

whole-body (18)F-FDG PET-MR followed by ileocolonoscopy within 24 h. Diagnostic

performance of five fecal biomarkers (calprotectin, lactoferrin,

polymorphonuclear leukocyte elastase, S100A12 and eosinophil-derived neurotoxin),

MR morphological parameters (MRmorph), diffusion-weighted imaging and PET in

detecting active disease determined by Rachmilewitz endoscopic activity index

(EAI) were evaluated and compared with each other. Correlations between fecal

biomarkers, PET and endoscopy were calculated. RESULTS: According to EAI, n = 38

patients presented with endoscopically active disease (16 mild, 19 moderate and 3

severe). All five biomarkers, PET and MRmorph could differentiate endoscopically

active disease from endoscopic remission without significant difference regarding

their operating characteristics (accuracies between 0.673 for calprotectin and

0.898 for lactoferrin). In predicting endoscopically moderate to severe disease,

PET showed the highest diagnostic performance (accuracy = 0.857) compared to

calprotectin and lactoferrin (accuracy = 0.633 and 0.735). PET had also the

strongest correlation with endoscopy (ρ = 0.685, p < 0.001), while within fecal

biomarkers the levels of lactoferrin and eosinophil-derived neurotoxin correlated

significantly with EAI (ρ = 0.423 and 0.528, both p < 0.05). CONCLUSION: Both

fecal biomarkers and PET-MR were excellent non-invasive diagnostic tools in the

assessment of disease activity in ulcerative colitis. ADVANCES IN KNOWLEDGE: Both

fecal biomarkers and PET-MR parameters are able to predict endoscopically active

disease with comparable diagnostic performance. PET had the highest correlation

with endoscopy and outperformed fecal biomarkers in differentiating moderate to

severe from mild disease.

FAU - Li, Yan

AU - Li Y

AUID- ORCID: 0000-0002-3906-9481

AD - Department of Diagnostic and Interventional Radiology and Neuroradiology,

University Hospital Essen, University of Duisburg-Essen, Hufelandstrasse 55,

45147 Essen, Germany.

FAU - Khamou, Michael

AU - Khamou M

AD - Department of Diagnostic and Interventional Radiology and Neuroradiology,

University Hospital Essen, University of Duisburg-Essen, Hufelandstrasse 55,

45147 Essen, Germany.

FAU - Schaarschmidt, Benedikt Michael

AU - Schaarschmidt BM

AD - Department of Diagnostic and Interventional Radiology and Neuroradiology,

University Hospital Essen, University of Duisburg-Essen, Hufelandstrasse 55,

45147 Essen, Germany.

FAU - Umutlu, Lale

AU - Umutlu L

AD - Department of Diagnostic and Interventional Radiology and Neuroradiology,

University Hospital Essen, University of Duisburg-Essen, Hufelandstrasse 55,

45147 Essen, Germany.

FAU - Forsting, Michael

AU - Forsting M

AD - Department of Diagnostic and Interventional Radiology and Neuroradiology,

University Hospital Essen, University of Duisburg-Essen, Hufelandstrasse 55,

45147 Essen, Germany.

FAU - Demircioglu, Aydin

AU - Demircioglu A

AD - Department of Diagnostic and Interventional Radiology and Neuroradiology,

University Hospital Essen, University of Duisburg-Essen, Hufelandstrasse 55,

45147 Essen, Germany.

FAU - Haubold, Johannes

AU - Haubold J

AD - Department of Diagnostic and Interventional Radiology and Neuroradiology,

University Hospital Essen, University of Duisburg-Essen, Hufelandstrasse 55,

45147 Essen, Germany.

FAU - Koch, Anna Katharina

AU - Koch AK

AD - Department of Internal and Integrative Medicine, Kliniken Essen-Mitte, University

of Duisburg-Essen, Am Deimelsberg 34a, 45276 Essen, Germany.

FAU - Bruckmann, Nils-Martin

AU - Bruckmann NM

AD - Department of Diagnostic and Interventional Radiology, University Hospital

Dusseldorf, Moorenstraße 5, 40225, Düsseldorf, Germany.

FAU - Sawicki, Lino Morris

AU - Sawicki LM

AD - Department of Diagnostic and Interventional Radiology, University Hospital

Dusseldorf, Moorenstraße 5, 40225, Düsseldorf, Germany.

FAU - Herrmann, Ken

AU - Herrmann K

AD - Department of Nuclear Medicine, University Hospital Essen, University of

Duisburg-Essen, Hufelandstrasse 55, 45147 Essen, Germany.

FAU - Boone, James Hunter

AU - Boone JH

AD - Research and Development, TechLab, INC., 2001 Kraft Drive, Blacksburg, USA.

FAU - Langhorst, Jost

AU - Langhorst J

AD - Department of Internal and Integrative Medicine, Kliniken Essen-Mitte, University

of Duisburg-Essen, Am Deimelsberg 34a, 45276 Essen, Germany.

AD - Department for Internal and Integrative Medicine, Social Foundation Bamberg,

Clinic Bamberg, Buger Straße 80, 96049 Bamberg, Germany.

AD - Chair for Integrative Medicine, University of Duisburg-Essen, Am Deimelsberg 34a,

45276 Essen, Germany.

LA - eng

SI - ClinicalTrials.gov/NCT03781284

PT - Comparative Study

PT - Journal Article

PT - Randomized Controlled Trial

DEP - 20200624

PL - England

TA - Br J Radiol

JT - The British journal of radiology

JID - 0373125

RN - 0 (Biomarkers)

RN - 0 (Leukocyte L1 Antigen Complex)

RN - 0 (S100A12 Protein)

RN - 0 (S100A12 protein, human)

RN - 0Z5B2CJX4D (Fluorodeoxyglucose F18)

RN - EC 3.1.- (Eosinophil-Derived Neurotoxin)

RN - EC 3.4.21.- (Lactoferrin)

RN - EC 3.4.21.37 (Leukocyte Elastase)

SB - IM

MH - Adult

MH - Aged

MH - Biomarkers/analysis

MH - Colitis, Ulcerative/*diagnosis/diagnostic imaging

MH - Colonoscopy

MH - Eosinophil-Derived Neurotoxin/analysis

MH - Feces/*chemistry

MH - Female

MH - Fluorodeoxyglucose F18

MH - Humans

MH - Lactoferrin/analysis

MH - Leukocyte Elastase/analysis

MH - Leukocyte L1 Antigen Complex/analysis

MH - Magnetic Resonance Imaging/*methods

MH - Middle Aged

MH - Multimodal Imaging/methods

MH - Positron-Emission Tomography/*methods

MH - S100A12 Protein/analysis

MH - Young Adult

PMC - PMC7446018

EDAT- 2020/06/25 06:00

MHDA- 2020/08/06 06:00

PMCR- 2021/08/01

CRDT- 2020/06/25 06:00

PHST- 2020/06/25 06:00 [pubmed]

PHST- 2020/08/06 06:00 [medline]

PHST- 2020/06/25 06:00 [entrez]

PHST- 2021/08/01 00:00 [pmc-release]

AID - 10.1259/bjr.20200167 [doi]

PST - ppublish

SO - Br J Radiol. 2020 Aug;93(1112):20200167. doi: 10.1259/bjr.20200167. Epub 2020 Jun

24.

PMID- 34399198

OWN - NLM

STAT- MEDLINE

DCOM- 20211124

LR - 20220531

IS - 1879-1360 (Electronic)

IS - 0022-3999 (Linking)

VI - 149

DP - 2021 Oct

TI - Mindfulness based cognitive therapy for youth with inflammatory bowel disease and

depression - Findings from a pilot randomised controlled trial.

PG - 110594

LID - S0022-3999(21)00239-7 [pii]

LID - 10.1016/j.jpsychores.2021.110594 [doi]

AB - BACKGROUND: Mindfulness-based cognitive therapy (MBCT) is a promising adjunctive

treatment for adolescents and young adults (AYAs) with Inflammatory Bowel Disease

(IBD) and comorbid depression. OBJECTIVES: This pilot randomised controlled trial

(RCT) aimed to evaluate feasibility and efficacy of an adapted MBCT program for

AYA, aged 16-29, with IBD. METHODS: Sixty-four AYAs were randomly allocated to

MBCT (n = 33) or treatment as usual (TAU) (n = 31). Primary outcome measure was

the depression score on Depression, Anxiety and Stress Scale. Secondary outcomes

included anxiety, stress, IBD-related quality of life, coping, mindfulness,

post-traumatic growth, medication adherence, IBD activity, inflammatory markers,

microbiome characteristics and brain functional connectivity. RESULTS: Study

recruitment rate was 75%, retention rate 70%, and session attendance 92%.

Intention to treat analyses revealed that, compared to TAU group, MBCT group had

significantly lower depression (∆ = -6.0; 95%CI = -10.8 to -1.2; P = 0.015) and

stress (∆ = -5.1; 95%CI = -10.1 to -0.0; P = 0.049), higher active coping

(∆ = 1.0;95%CI = 0.1-1.9; P = 0.022), and total mindfulness scores

(∆ = 10.9;95%CI = 1.1-20.8; P = 0.030) at 8 weeks (post-therapy), and improved

coping by positive reframing (∆ = 1.1;95%CI = 0.0-2.2; P = 0.043) and planning

(∆ = 0.9;95%CI = 0.0-1.9; P = 0.045), mindful awareness

(∆ = 5.2.;95%CI = 2.0-8.5; P = 0.002) and total mindfulness scores

(∆ = 10.8.;95%CI = 0.4-21.1; P = 0.042) at 20 weeks. On per protocol analysis,

MBCT group had significantly lower depression (∆ = -6.3; 95%CI = -11.4 to -1.2;

P = 0.015), stress (∆ = -6.0; 95%CI = -11.2 to -0.5; P = 0.032), increased active

coping (∆ = 0.9;95%CI = 0-1.7; P = 0.05) at 8 weeks, and mindful awareness

(∆ = 5.4; 95%CI = 2.1-8.6; P = 0.001) at 20 weeks. CONCLUSION: In AYAs with IBD,

MBCT is feasible and beneficial in improving depression, stress, mindfulness and

adaptive coping. It holds promise as an important component of integrated IBD

care. Trial registration number ACTRN12617000876392, U1111-1197-7370;

Pre-results.

CI - Copyright © 2021 Elsevier Inc. All rights reserved.

FAU - Ewais, T

AU - Ewais T

AD - School of Medicine, Mater Clinical School and Princess Alexandra Clinical School,

Raymond Terrace, The University of Queensland, South Brisbane, Queensland 4101,

Australia; Mater Young Adult Health Centre, Mater Misericordiae Ltd, Raymond

Terrace, South Brisbane, Queensland 4101, Australia; Mater Research Institute,

Mater Misericordiae Ltd, Raymond Terrace, South Brisbane, Queensland 4101,

Australia; School of Medicine, Griffith University, Gold Coast, Queensland 5005,

Australia. Electronic address: t.ewais@uq.edu.au.

FAU - Begun, J

AU - Begun J

AD - School of Medicine, Mater Clinical School and Princess Alexandra Clinical School,

Raymond Terrace, The University of Queensland, South Brisbane, Queensland 4101,

Australia; Mater Young Adult Health Centre, Mater Misericordiae Ltd, Raymond

Terrace, South Brisbane, Queensland 4101, Australia; Mater Research Institute,

Mater Misericordiae Ltd, Raymond Terrace, South Brisbane, Queensland 4101,

Australia. Electronic address: Jakob.Begun@mater.uq.edu.au.

FAU - Kenny, M

AU - Kenny M

AD - The University of Adelaide, Adelaide, South Australia 5005, Australia. Electronic

address: Maura.Kenny@sa.gov.au.

FAU - Hay, K

AU - Hay K

AD - QIMR Berghofer Medical Research Institute, 300 Herston Road, Queensland 4006,

Australia. Electronic address: k.hay@qimrberghofer.uq.edu.au.

FAU - Houldin, Evan

AU - Houldin E

AD - Queensland Brain Institute, St Lucia, Queensland 4072, Australia. Electronic

address: e.houldin@uq.edu.au.

FAU - Chuang, Kai-Hisang

AU - Chuang KH

AD - Queensland Brain Institute, St Lucia, Queensland 4072, Australia. Electronic

address: k.chuang@uq.edu.au.

FAU - Tefay, M

AU - Tefay M

AD - Mater Young Adult Health Centre, Mater Misericordiae Ltd, Raymond Terrace, South

Brisbane, Queensland 4101, Australia. Electronic address:

Merilyn.tefay@mater.org.au.

FAU - Kisely, S

AU - Kisely S

AD - School of Medicine, Mater Clinical School and Princess Alexandra Clinical School,

Raymond Terrace, The University of Queensland, South Brisbane, Queensland 4101,

Australia; Metro South Health Service, Wooloongabba, Queensland 4102, Australia;

Departments of Psychiatry, Community Health and Epidemiology, Dalhousie

University, Halifax, Nova Scotia, Canada. Electronic address: s.kisely@uq.edu.au.

LA - eng

GR - K23 AT011173/AT/NCCIH NIH HHS/United States

PT - Journal Article

PT - Randomized Controlled Trial

PT - Research Support, N.I.H., Extramural

DEP - 20210802

PL - England

TA - J Psychosom Res

JT - Journal of psychosomatic research

JID - 0376333

SB - IM

MH - Adolescent

MH - Adult

MH - *Cognitive Behavioral Therapy

MH - Depression/therapy

MH - Humans

MH - *Inflammatory Bowel Diseases/therapy

MH - *Mindfulness

MH - Pilot Projects

MH - Treatment Outcome

MH - Young Adult

OTO - NOTNLM

OT - Adolescents and young adults

OT - Depression

OT - IBD

OT - Mindfulness

EDAT- 2021/08/17 06:00

MHDA- 2021/11/25 06:00

CRDT- 2021/08/16 20:14

PHST- 2021/02/22 00:00 [received]

PHST- 2021/07/21 00:00 [revised]

PHST- 2021/07/31 00:00 [accepted]

PHST- 2021/08/17 06:00 [pubmed]

PHST- 2021/11/25 06:00 [medline]

PHST- 2021/08/16 20:14 [entrez]

AID - S0022-3999(21)00239-7 [pii]

AID - 10.1016/j.jpsychores.2021.110594 [doi]

PST - ppublish

SO - J Psychosom Res. 2021 Oct;149:110594. doi: 10.1016/j.jpsychores.2021.110594. Epub

2021 Aug 2.

PMID- 38154825

OWN - NLM

STAT- MEDLINE

DCOM- 20240103

LR - 20240112

IS - 2054-4774 (Print)

IS - 2054-4774 (Electronic)

IS - 2054-4774 (Linking)

VI - 10

IP - 1

DP - 2023 Dec 28

TI - People with IBD evidence more microarousals during sleep architecture

assessments.

LID - 10.1136/bmjgast-2023-001249 [doi]

LID - e001249

AB - OBJECTIVE: Poor sleep is common in inflammatory bowel disease (IBD) and may be

associated with overall worse disease outcomes. While the sleep/IBD literature is

growing, the data are often self-reported. Further, much of the research using

objective measures of sleep architecture, or the overall pattern of sleep depth,

rely on single-night assessments, which can be of questionable validity. DESIGN:

Participants with IBD and healthy controls were recruited from

Dartmouth-Hitchcock Medical Center as part of a two-phase clinical trial. Sleep

architecture was assessed using three nights of in-home electroencephalographic

monitoring and scored according to the American Academy of Sleep Medicine

guidelines. RESULTS: Our sample included 15 participants with IBD and 8 healthy

controls. Participants with IBD were more psychiatrically complex, with more

self-reported insomnia, anxiety and depression. Participants with IBD evidenced

greater microarousals than healthy controls. In participants with IBD,

microarousals were associated with lower insomnia and greater depression scores.

Within IBD, participants with clinically significant insomnia evidenced trend

towards lower sleep efficiency, while self-reported disease activity did not

significantly impact findings. CONCLUSIONS: The methodology of past research may

have impacted findings, including the reliance on single-night assessments and

limited generalisability. Future research that uses robust, multinight

assessments of sleep architecture in large, diverse samples is clearly warranted,

as is research exploring the impact of cognitive and behavioural factors on sleep

architecture and arousal. TRIAL REGISTRATION NUMBER: NCT04132024.

CI - © Author(s) (or their employer(s)) 2023. Re-use permitted under CC BY-NC. No

commercial re-use. See rights and permissions. Published by BMJ.

FAU - Salwen-Deremer, Jessica K

AU - Salwen-Deremer JK

AUID- ORCID: 0000-0001-6498-3729

AD - Department of Psychiatry, Dartmouth-Hitchcock Medical Center, Lebanon, New

Hampshire, USA jessica.k.salwen-deremer@hitchcock.org.

AD - Center for Digestive Health, Dartmouth-Hitchcock Medical Center, Lebanon, New

Hampshire, USA.

FAU - Reid, Matthew J

AU - Reid MJ

AD - Department of Psychiatry and Behavioral Sciences, Johns Hopkins School of

Medicine, Baltimore, Maryland, USA.

FAU - Westvold, Sarah J

AU - Westvold SJ

AD - Center for Digestive Health, Dartmouth-Hitchcock Medical Center, Lebanon, New

Hampshire, USA.

AD - Yale School of Medicine, New Haven, Connecticut, USA.

FAU - Siegel, Corey A

AU - Siegel CA

AD - Center for Digestive Health, Dartmouth-Hitchcock Medical Center, Lebanon, New

Hampshire, USA.

FAU - Smith, Michael T

AU - Smith MT

AD - Department of Psychiatry and Behavioral Sciences, Johns Hopkins School of

Medicine, Baltimore, Maryland, USA.

LA - eng

SI - ClinicalTrials.gov/NCT04132024

PT - Clinical Trial

PT - Journal Article

PT - Research Support, Non-U.S. Gov't

DEP - 20231228

PL - England

TA - BMJ Open Gastroenterol

JT - BMJ open gastroenterology

JID - 101660690

SB - IM

MH - Humans

MH - Anxiety

MH - *Inflammatory Bowel Diseases/complications/epidemiology/psychology

MH - Self Report

MH - Sleep

MH - *Sleep Initiation and Maintenance Disorders/epidemiology

PMC - PMC10759128

OTO - NOTNLM

OT - CROHN'S DISEASE

OT - PSYCHOLOGY

OT - ULCERATIVE COLITIS

COIS- Competing interests: None declared.

EDAT- 2023/12/29 00:42

MHDA- 2024/01/03 08:01

PMCR- 2023/12/28

CRDT- 2023/12/28 20:53

PHST- 2023/09/07 00:00 [received]

PHST- 2023/11/29 00:00 [accepted]

PHST- 2024/01/03 08:01 [medline]

PHST- 2023/12/29 00:42 [pubmed]

PHST- 2023/12/28 20:53 [entrez]

PHST- 2023/12/28 00:00 [pmc-release]

AID - bmjgast-2023-001249 [pii]

AID - 10.1136/bmjgast-2023-001249 [doi]

PST - epublish

SO - BMJ Open Gastroenterol. 2023 Dec 28;10(1):e001249. doi:

10.1136/bmjgast-2023-001249.
